# Total Synthesis, Structure, and Biological Activity of Adenosylrhodibalamin, the Non‐Natural Rhodium Homologue of Coenzyme B_12_

**DOI:** 10.1002/anie.201603738

**Published:** 2016-06-29

**Authors:** Florian J. Widner, Andrew D. Lawrence, Evelyne Deery, Dana Heldt, Stefanie Frank, Karl Gruber, Klaus Wurst, Martin J. Warren, Bernhard Kräutler

**Affiliations:** ^1^ Institut für Organische Chemie und Centrum für Molekulare Biowissenschaften (CMBI)Universität Innsbruck6020InnsbruckAustria; ^2^ School of BiosciencesUniversity of KentCanterburyCT2 7NJUK; ^3^ Institut für Molekulare BiowissenschaftenUniversität GrazAustria; ^4^ Institut für Allgemeine, Anorganische und Theoretische ChemieUniversität InnsbruckAustria; ^5^ Plant and Microbial Biology DepartmentUniversity of CaliforniaBerkeleyCAUSA

**Keywords:** biosynthesis, cobalt, total synthesis, transition metal, Vitamin B_12_

## Abstract

B_12_ is unique among the vitamins as it is biosynthesized only by certain prokaryotes. The complexity of its synthesis relates to its distinctive cobalt corrin structure, which is essential for B_12_ biochemistry and renders coenzyme B_12_ (AdoCbl) so intriguingly suitable for enzymatic radical reactions. However, why is cobalt so fit for its role in B_12_‐dependent enzymes? To address this question, we considered the substitution of cobalt in AdoCbl with rhodium to generate the rhodium analogue 5′‐deoxy‐5′‐adenosylrhodibalamin (AdoRbl). AdoRbl was prepared by de novo total synthesis involving both biological and chemical steps. AdoRbl was found to be inactive in vivo in microbial bioassays for methionine synthase and acted as an in vitro inhibitor of an AdoCbl‐dependent diol dehydratase. Solution NMR studies of AdoRbl revealed a structure similar to that of AdoCbl. However, the crystal structure of AdoRbl revealed a conspicuously better fit of the corrin ligand for Rh^III^ than for Co^III^, challenging the current views concerning the evolution of corrins.

The biochemical activity of the biological forms of B_12_ is based on the pivotal role played by the cobalt center bound by the corrin ring.^1^ However, why is cobalt, rather than any other metal, so suited to its role in B_12_?1b This old question has posed a formidable challenge.^1^, ^2^ Interestingly, cobalt was given its name because German miners found it in ores contaminated with arsenic, and believed it was added malevolently by “Kobolds”, or goblins. To address the “cobalt question”, we considered the replacement of cobalt by its heavier Group IX homologue rhodium. The specific suitability of coenzyme B_12_ (5′‐deoxy‐5′‐adenosylcobalamin, AdoCbl; Figure 1) as a catalytic radical source by enzyme‐controlled homolytic cleavage of its Co−C bond^3^ suggested that its rhodium homologue, 5′‐deoxy‐5′‐adenosyl‐rhodibalamin (AdoRbl), would be a particularly interesting target. AdoRbl was first prepared in the 1970s via metal‐free hydrogenobalamin, which was isolated in low yields from cultures of *Chromatium vinosum* grown in cobalt‐free media, but incompletely characterized.2a Unfortunately, various alternative strategies to generate metal analogues of the natural corrinoids by removal of the Co center of vitamin B_12_ derivatives have not been successful (see e.g. Ref. 4); therefore, a novel approach for its preparation was required. Herein, we describe a concise total synthesis of AdoRbl through a strategical combination of biological and chemical means, and report its structural and basic biological properties. Indeed, as described below, by asking “Why not rhodium?”, we have addressed a related fundamental question concerning the evolutionary selection and adaptation of corrins.

**Figure 1:**
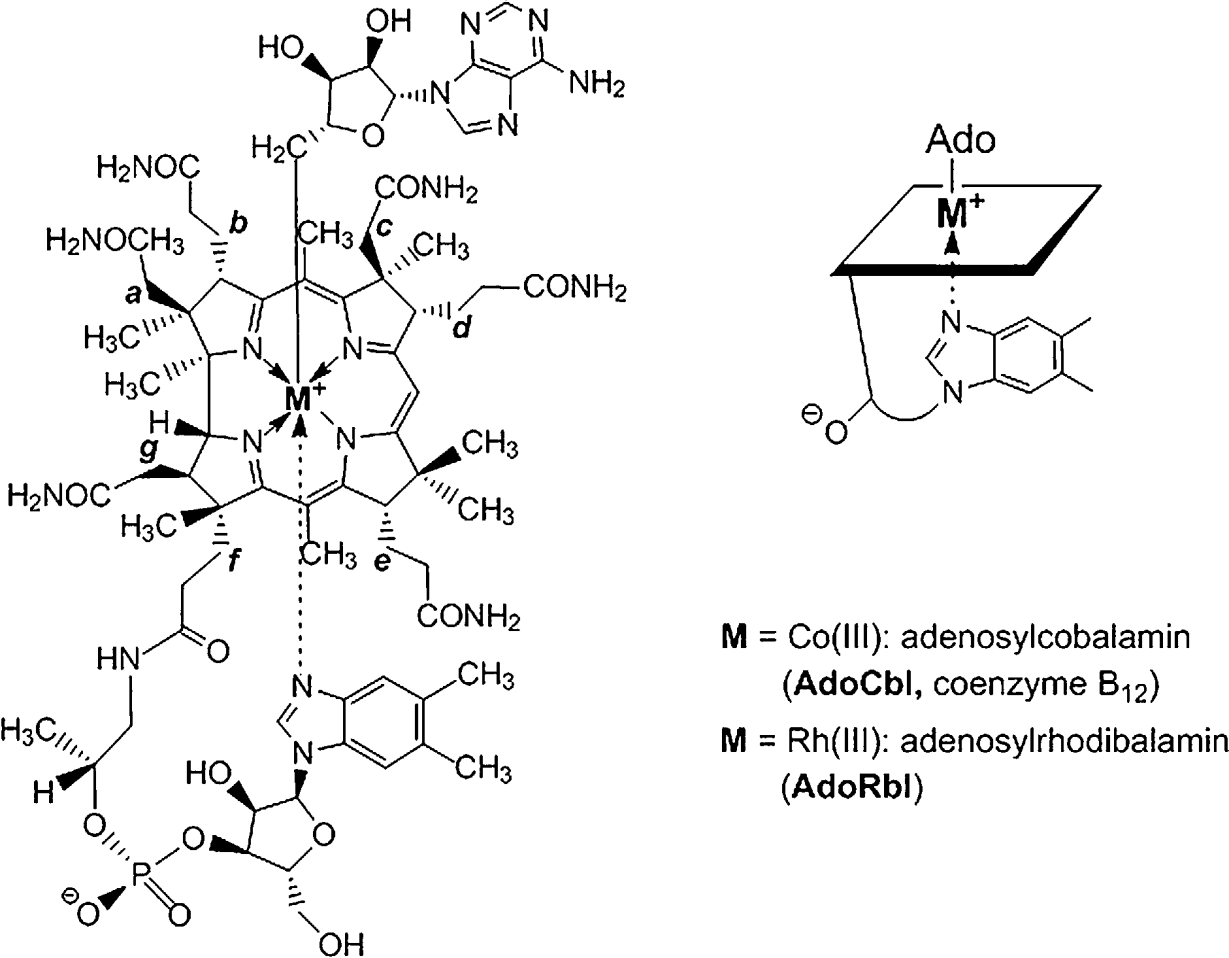
Chemical formula of coenzyme B_12_ (M=Co^III^, AdoCbl) and 5′‐deoxy‐5′‐adenosylrhodibalamin (M=Rh^III^, AdoRbl).

Complementary chemical and biological methods were developed for the synthesis of 5′‐deoxy‐5′adenosylrhodibalamin (AdoRbl; Figure 2). Initially, hydrogenobyrinic acid *a*,*c*‐diamide (Hbad) was synthesized de novo and in vivo using an engineered *E. coli* strain containing the ten genes (*cobA*‐*I*‐*G*‐*J*‐*M*‐*F*‐*K*‐*L*‐*H*‐*B*) that encode the enzymes for the biosynthesis of cobalamin from the endogenous biosynthetic intermediate uroporphyrinogen III.^5^ From around 30 L of culture, 88.2 mg of Hbad were obtained. Hbad was converted into rhodibyrinic acid *a*,*c*‐diamide by chemical insertion of Rh^I^.^6^ Orange‐red dicyanorhodi(III)byrinic acid *a*,*c*‐diamide (CN_2_‐Rhbad)6b was obtained in 75 % yield, but unfortunately proved to be resistant to refunctionalization into adenosylrhodi(III)byrinic acid *a*,*c*‐diamide (AdoRhbad). However, AdoRhbad was synthetically accessible when rhodibyrinic acid *a*,*c*‐diamide was isolated as the dichloro‐substituted Rh^III^ corrinoid (DCRhbad), which was characterized by UV/Vis spectroscopy and mass spectrometry. Reduction of DCRhbad with sodium borohydride in deoxygenated solution led to the light yellow Rh^I^ corrinoid. Treatment of the latter with 5′‐iodo‐5′‐deoxyadenosine (0 °C→RT) gave an orange reaction mixture from which AdoRhbad could be isolated in 75 % yield. The molecular formula of AdoRhbad was confirmed by ESI mass spectrometry (*m*/*z* 1230.3 [*M*]^+^). Its UV/Vis spectrum was similar to those of the dichloro and dicyano Rh^III^ analogues (see the Supporting Information, Figure S1). The metal‐bound methylene group of the 5′‐deoxyadenosyl moiety of AdoRhbad gave rise to two characteristic multiplets at high field in the ^1^H NMR spectrum, which were assigned to the diastereotopic protons of this CH_2_ group (Figure S2). Both of these resonances of AdoRhbad showed a diagnostic 1.7 Hz coupling with ^103^Rh (*I*=1/2).

**Figure 2:**
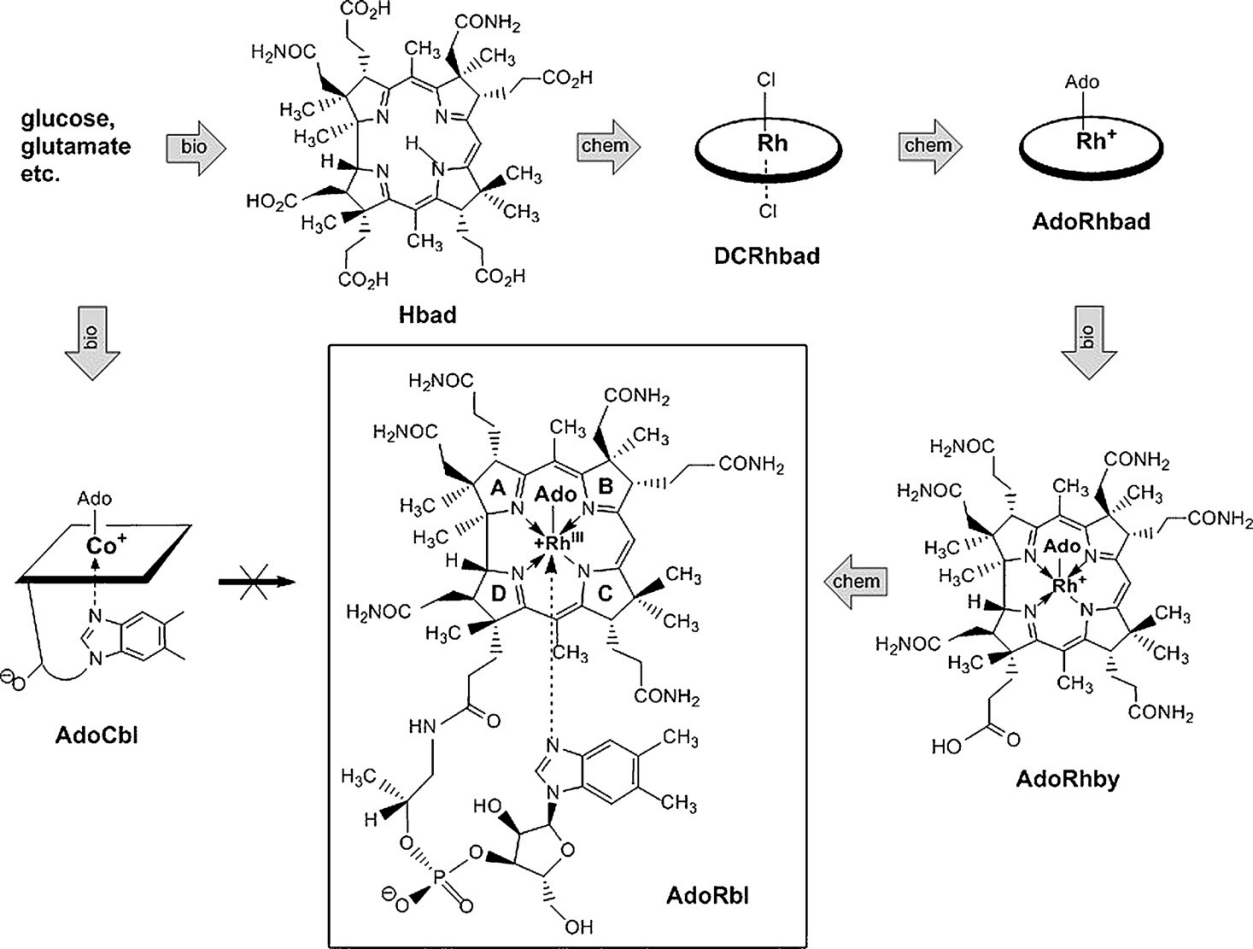
Total synthesis of 5′‐deoxy‐5′‐adenosyl‐rhodi(III)balamin (AdoRbl) by intertwined enzymatic steps of the machinery for the biosynthesis of AdoCbl (bio) and chemical steps (chem) in the sequence bio‐chem‐chem‐bio‐chem. AdoRbl cannot be made from AdoCbl.

With the Rh analogue of adenosylcobyrinic acid *a*,*c*‐diamide in hand, the cobalamin (Cbl) biosynthetic pathway was again employed, namely in the form of CobQ,^7^ to specifically amidate four of the remaining five side‐chain carboxyl groups, thus converting AdoRhbad into adenosylrhodi(III)byric acid (AdoRhby; see Figure 2). Indeed, AdoRhbad served remarkably well as a pseudo‐substrate for CobQ and furnished AdoRhby in 92 % yield. The regiospecific fourfold amidation of the peripheral side chains was confirmed by ESI mass spectrometry and ^1^H NMR spectroscopy.

5′‐Deoxy‐5′‐adenosylrhodibalamin (AdoRbl), the Rh analogue of coenzyme B_12_ (AdoCbl), was prepared by chemical conjugation of AdoRhby with the B_12_ nucleotide moiety.1a, 8 This was achieved by activation of AdoRhby with the carbodiimide reagent 1‐ethyl‐3‐(3‐dimethylaminopropyl)carbodiimide (EDC) in the presence of the B_12_ nucleotide, furnishing the orange‐red AdoRbl in 79 % yield. The UV/Vis spectrum of isolated AdoRbl showed absorption maxima at *λ*=512, 491, and 350 nm (Figure S1), as reported previously.^2^ The spectrum of AdoRbl is surprisingly similar to that of cyanocobalamin (vitamin B_12_), but differs significantly from that of coenzyme B_12_ (AdoCbl). As also noted earlier,^3^ AdoRbl does not decompose when exposed to daylight in aerated solutions, in contrast to the photosensitive AdoCbl.^9^

The 500 MHz ^1^H NMR spectrum of AdoRbl in D_2_O contained resonances for all carbon‐bound H atoms, including the characteristic doublet‐ and triplet‐like signals at high field for the Rh‐bound CH_2_ group of the Ado ligand (Figure 3). This latter finding is in striking contrast to the spectrum reported in the earlier work on AdoRbl, where such signals were not recorded.^2^ The high‐field resonances of the diastereotopic protons H_A_C5RL and H_B_C5RL of AdoRbl correspond to similar resonances in the spectrum of AdoCbl, identified there as H_re_ and H_si_, respectively, but both are shifted upfield by about *δ*=1.31 ppm. The crystal structure of AdoRbl exhibits an antiperiplanar arrangement of HC4RL and of H_re_ at C5RL, also supporting the assignment of the triplet‐like resonance of H_A_C5RL to H_re_. ^1^H,^1^H NOE spectra (Figures S3 and S4) helped to confirm the base‐on nature of AdoRbl and the attachment of the 5′‐deoxyadenosyl moiety at the β‐side of the corrin‐bound Rh center. The 2D NMR data provided further characteristic chemical shift information (e.g., for the protons in the side chains of ring B; see Tables S1–S3), indicating similar conformations of the corrin ligand and both axially bound groups for AdoRbl and AdoCbl. The structures of AdoRbl and coenzyme B_12_ (AdoCbl)^10^ in aqueous solution are therefore very similar.

**Figure 3:**
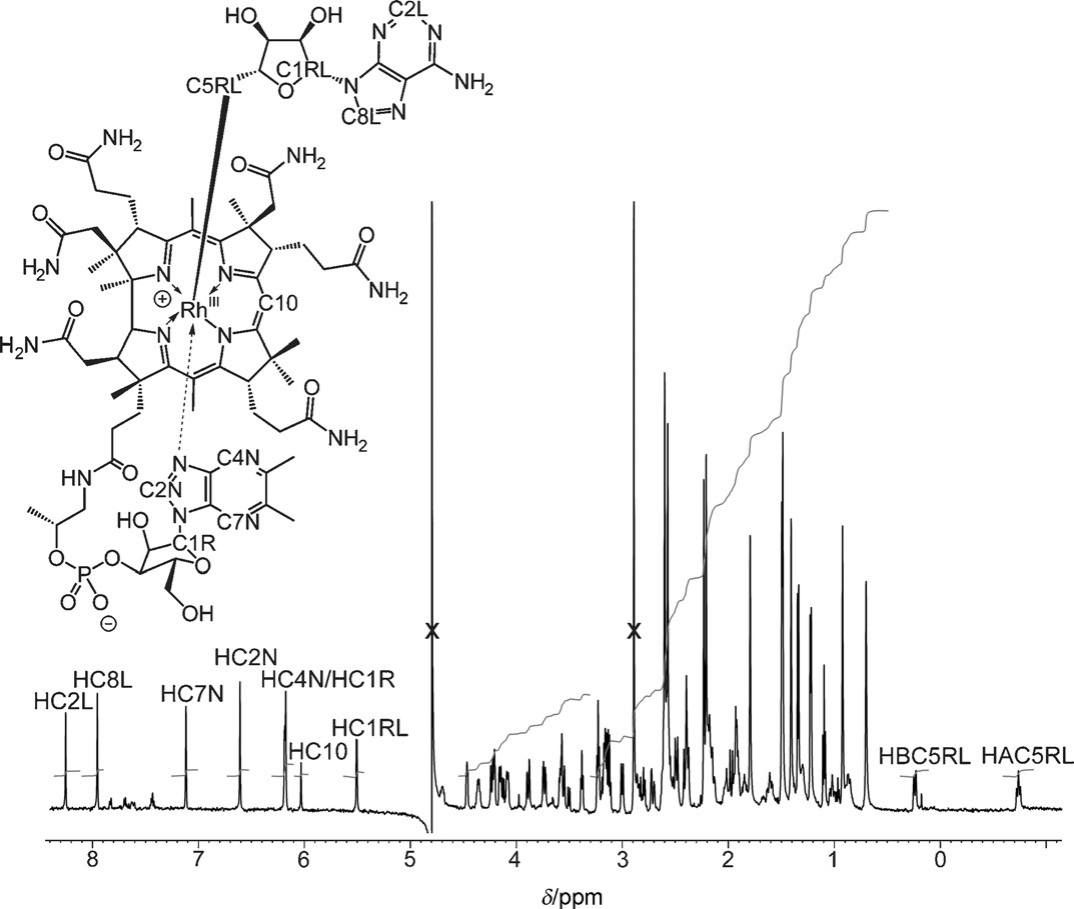
500 MHz ^1^H NMR spectrum of AdoRbl (0.4 mm in D_2_O, 10 mm potassium phosphate, pD 7.4, 298 K, suppressed HDO signal).

AdoRbl crystallized from a solution of water/acetonitrile as dark red monoclinic prisms (space group *C*2, No.5). A well resolved X‐ray crystal structure was obtained for AdoRbl, which is the first of any metal analogue of the Cbl series. It confirmed the NMR‐derived chemical constitution of AdoRbl and showed basic structural features similar to those of AdoCbl^11^ (Figure 4 and Figure S6). As in AdoCbl, the Ado group of AdoRbl is positioned roughly above ring C of the corrin ring. However, in AdoRbl, it has been rotated counterclockwise by about 24° (Figure S7), and is held in position by an unprecedented hydrogen‐bonded dimerization interface involving the Ado moiety and both of the side chains of ring B (Figure S9). Thus the Ado group is closer to ring B in AdoRbl than in AdoCbl,^11^ and ring B adopts a conformation unparalleled in Cbls (see below).

**Figure 4:**
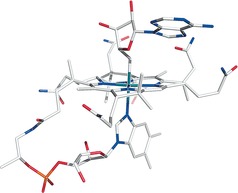
Stick model of adenosylrhodibalamin (AdoRbl) from the crystal structure. C gray, N blue, O red, P orange, Rh blue‐green. For details, see Figures S6–S8.

The biological activity of AdoRbl as an AdoCbl analogue was initially investigated in the form of a bioassay^12^ by monitoring the activity of methionine synthase (MetH), and subsequently by a direct enzymatic assay with diol (propanediol) dehydratase (PduCDE). For MetH, which utilizes a B_12_ cofactor, we employed a plate‐based microbiological bioassay that uses a *Salmonella enterica cbiB metE* reporter strain that is reliant upon exogenous cobalamin (Cbl) for its MetH when grown on minimal media. The size of the growth circles observed on these plates is related logarithmically to the quantity of applied Cbl (Figure 5).

**Figure 5:**
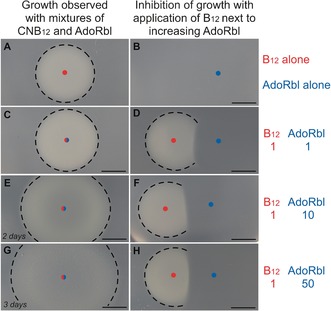
AdoRbl in a microbial bioassay. Application of vitamin B_12_ (CNCbl) to the plate promotes the growth of a *S. enterica cbiB metE* strain (A). AdoRbl on its own does not promote growth (B). Mixing AdoRbl with B_12_ results in increased growth circles that are noticeably less dense than the B_12_ growth halos (C, E, G). Addition of AdoRbl in close proximity to the point of application of B_12_ results in the appearance of a zone of inhibition that gets more extensive as more AdoRbl is added (D, F, H).

Addition of AdoRbl alone to the bioassay plates did not promote any growth. However, when AdoRbl was applied in close proximity to an equivalent amount of vitamin B_12_ (CNCbl), a growth inhibition zone around the AdoRbl application point was observed. Increasing the concentration of AdoRbl resulted in greater inhibition (Figure 5). Unexpectedly, a mixture of CNCbl and AdoRbl resulted in a larger but more diffuse growth circle. These observed growth patterns indicate that 1) AdoRbl is not converted into an active cofactor form for methionine synthase, and that 2) AdoRbl acts as an inhibitor for Cbl either by preventing the uptake of Cbl from the medium or by competing for the active site of methionine synthase. Indeed, the larger growth circles that were observed when CNCbl was mixed with an excess of AdoRbl can be explained best by the ability of this analogue to actively interact with the regulation of Cbl uptake through a B_12_ riboswitch.^13^ In *E. coli* and *S. enterica*, the *btuB* riboswitch acts as a feedback control mechanism, with AdoCbl as the preferred ligand,^13^, ^14^ to switch off the production of the outer‐membrane B_12_ transporter. The increased growth circles on the bioassay plates are consistent with AdoRbl reducing the level of Cbl uptake.

The effect of AdoRbl on the activity of AdoCbl‐dependent enzymes was investigated by studying the *Citrobacter freundii* 1,2‐propanediol dehydratase (Figures S10–S12). The kinetic constants for the reaction catalyzed by purified 1,2‐propanediol dehydratase were determined by non‐linear regression. The enzyme was found to be inactive with AdoRbl as a pseudo‐coenzyme. However, in the presence of AdoCbl, the enzyme was active, with a *K*_m_ value of 3.0 μm for AdoCbl and *k*_cat_=358 s^−1^ (based on an α_2_β_2_γ_2_ quaternary structure). Both AdoRbl and vitamin B_12_ were found to be competitive inhibitors of the enzyme, with *K*_i_ values of 6.9 μm for AdoRbl and 2.5 μm for vitamin B_12_. These results confirm that AdoRbl acts as an inhibitory analogue of AdoCbl and is unable to catalyze the propanediol dehydratase reaction.

The biological roles of cobalt^15^ and the functional forms of B_12_ 16 appear to be largely interdependent and remarkably exclusive. Thus the question of why cobalt, rather than any other transition metal, is found in B_12_ has spurred interest in developing metal analogues of B_12_.^6^ In this respect, the Group IX metal rhodium represents a prime substitute. We thus developed a strategy for the total synthesis of 5′‐deoxy‐5′‐adenosylrhodibalamin (AdoRbl), which is based on a concise sequence of biological and chemical steps. Structural studies with AdoRbl in solution (by NMR) and in the crystal confirmed the expected structural similarity to AdoCbl. However, the crystal structure of AdoRbl also revealed some remarkable consequences when Co^III^ is replaced with a larger Rh^III^ ion in AdoRbl. As expected, all six bonds to the metal center of AdoRbl were longer than those in AdoCbl, which is consistent with the 0.06 Å larger covalent radius of Rh.^17^ In fact, the four equatorial bonds were found to be elongated by 0.082(5) Å and the axial bonds by only 0.035(5) Å compared to those in AdoCbl (Figure 6).

**Figure 6:**
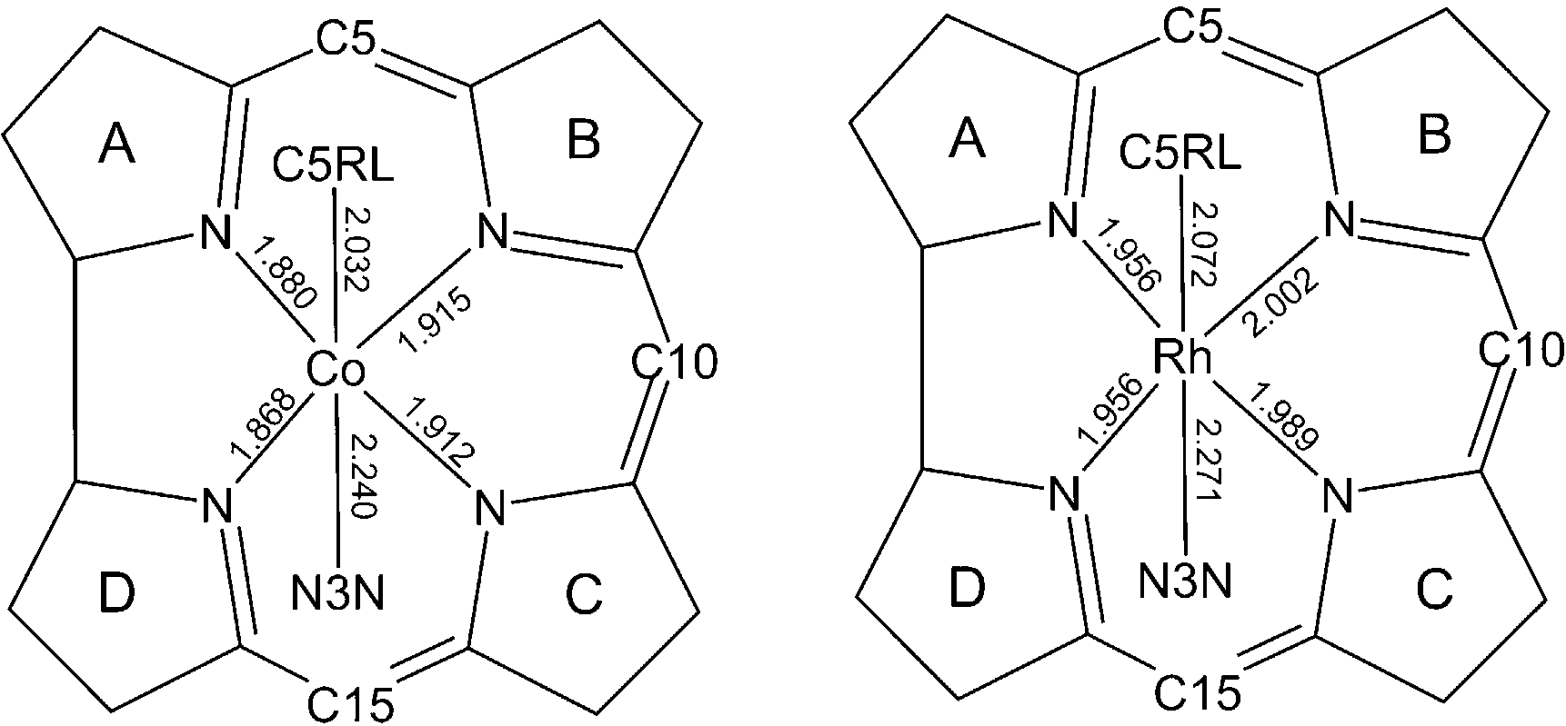
Comparison of the crystal structures of adenosylrhodibalamin (AdoRbl) and coenzyme B_12_ (AdoCbl). Bond lengths (in Å) to Co^III^ in AdoCbl and to Rh^III^ in AdoRbl.

Furthermore, the flatter corrin ligand of AdoRbl displays a record small fold angle of 5.9(2)° (13.3° in AdoCbl; see Figures 7, S8, and S9).^18^ Ring B of AdoRbl exhibits a striking reversed conformational twist when compared to the structures of AdoCbl and other natural corrinoids. Ring B of AdoRbl is also flattened, and its acetamide and propionamide substituents are both in a pseudo‐equatorial position. In contrast, the NMR solution structure indicated a ring B conformation in AdoRbl matching that in AdoCbl,^10^ and revealed no sign of the unprecedented “reversed” twist. Thus ring B of AdoRbl is, in fact, flexible and undergoes conformational inversion in the crystal.

**Figure 7:**
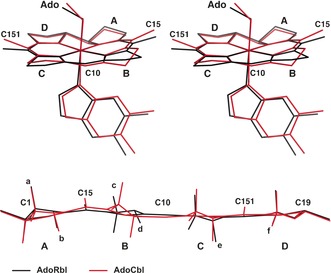
Comparison of the crystal structures of adenosylrhodibalamin (AdoRbl, black lines) and coenzyme B_12_ (AdoCbl, red lines). Top: Stereoview of the superimposed structures of AdoRbl and AdoCbl, highlighting the different folding of the corrin core. Bottom: Superposition of the corrin cores of AdoRbl and AdoCbl in cylindrical projections, showing the stronger folding of the corrin ligand of AdoCbl compared to that of AdoRbl as well as the conformational inversion of ring B (small letters: side chains; capital letters: rings of the corrin ligand). See the Supporting Information for details.

Hence, a comparison of the AdoRbl and AdoCbl structures reveals that, counterintuitively, the larger Rh^III^ ion fits better into the corrin ligand than the biologically relevant Co^III^ ion. Eschenmoser and Kratky have analyzed the fundamental structural consequences of a mutual misfit between small coordinated metal ions (e.g., low‐spin Ni^II^) and the coordination hole of tetrapyrrolic macrocycles.1a, 19 They suggested that contraction of the macrocycle leads to non‐planar (“saddle‐shaped”) porphyrins and a correlated conformational change of the four pyrrolic rings. Similar conformational effects of the mutual misfit of the size of the metal ion and the coordination hole have been observed for a range of porphyrinoid metal complexes^20^ and have been recognized to be an important factor in modifying the biological activity of metal porphyrinoids.^21^

The crystal structure of AdoRbl indicates that the coordination hole of the corrin ligand is slightly too large for the coordination of Co^III^ ions. In fact, in natural Co^III^ corrinoids, a significant corrin fold (13.3° in AdoCbl) is also apparent, as are notable twists of all four pyrrolic rings^22^ (Figure S8). These twists are most prominent in ring B, where a consistent conformational twist “in phase” with the non‐planar corrin macrocycle is observed.1a, 22a In contrast, the crystallographic studies with AdoRbl suggest a significant conformational relaxation of the corrin macrocycle when adapting its structure to the coordination requirements of Rh^III^.

Nature has evolved the unique “constitutional ring contraction” of the corrin ligand1a, 23 to reduce its hole size and to accommodate cobalt.^24^ However, as discussed here, the structural data are consistent with an additional conformational adaptation of the corrin ligand to meet the effective size of the coordinated Co^III^ ions. Hence, the observed better fit of Rh^III^ over Co^III^ suggests that the corrin ligand of cobalamin may not primarily be targeted by Nature to Co^III^. As rhodium is not considered to be an element that is essential for life on Earth,^15^ the interaction of the corrin ligand with Co, rather than Rh, ions deserves close attention, including reduced Co^II^ and Co^I^ forms. AdoCbl and cob(II)alamin feature very similar cobalt corrin structures,^25^ but crystallographic data of a Co^I^ corrin are not yet available (see, for example, Ref. 16a). A slight expansion of the coordination hole of the corrin ligand has been calculated to assist the reduction of Co^III^ and Co^II^ corrins.^26^ It is thus tempting to suggest that corrins may display a particular fit and stabilization for the polarizable Co^I^ ions, the action center of the enigmatic “supernucleophilic” Co^I^ corrins.1b, 16a,16b, 27 By analogy with the idea of enzymes evolving to stabilize a transition state to lower the activation energy of the reaction,^28^ the proposed ability of stabilizing the Co^I^ state over Co^III^ and Co^II^ forms would be a crucial aspect of the corrin ligand in enzyme reactions with Co^I^ corrin intermediates,^26^, ^27^, ^29^ which are difficult to generate in a biological environment.^14^, ^29^ This property would have allowed the selection of B_12_ to be fine‐tuned for its role as an essential organometallic catalyst for the prebiotic chemistry of life,^30^ in line with the proposed antiquity of cobalt corrins as ancient cofactors.1a

The molecular recipe for the biosynthesis of coenzyme B_12_ (AdoCbl) is confined to the genomes of only certain prokaryotes.^31^ By combining it with an engineered *E*. *coli* strain, a concise biological/chemical synthesis pathway to AdoRbl became available. AdoRbl was characterized as a structural, but not functional, mimic of the B_12_ coenzyme AdoCbl. The coenzyme inactivity of the largely isostructural Rh analogue of coenzyme B_12_, in combination with the inhibitory action of AdoRbl, suggests inefficient Rh−C bond homolysis of the enzyme‐bound AdoRbl. The determination^32^ of the strength of the Rh−C bond in AdoRbl will provide an experimental test for this conclusion.

Having re‐addressed the fundamental question of “Why cobalt?”,^1^ perhaps we should now ask: “Why not rhodium or another metal?” Metal analogues of the cobalamins (metbalamins) are believed to be inactive as cofactors, which is consistent with our studies on AdoRbl. Indeed, some metbalamins have been shown to inhibit bacterial growth.^6^ Suitably structured metbalamins may thus represent effective B_12_ antimetabolites or “antivitamins B_12_”,^33^ which are of growing interest in view of recent detailed structural studies concerning remarkable “novel” biological functions of Cbls.^34^ Our combined biological and chemical synthesis approach to the “rhodium problem” has opened a new entry to metbalamins and other metallocorrinoids, an exciting though poorly explored territory in the multifaceted B_12_ field.

## Experimental Section

See the Supporting Information for materials, instruments, strains used, construction of plasmids, details of synthetic and enzymatic procedures, spectroscopy, and X‐ray crystallography.

X‐ray crystallography: CCDC 1450631 (AdoRbl) contains the supplementary crystallographic data for this paper. These data are provided free of charge by The Cambridge Crystallographic Data Centre.

*Dedicated to Professor Albert Eschenmoser on the occasion of his 91st birthday*

## Supporting information

As a service to our authors and readers, this journal provides supporting information supplied by the authors. Such materials are peer reviewed and may be re‐organized for online delivery, but are not copy‐edited or typeset. Technical support issues arising from supporting information (other than missing files) should be addressed to the authors.

Supplementary
