# Supplementary material for: Total Synthesis, Structure, and Biological Activity of Adenosylrhodibalamin, the Non‐Natural Rhodium Homologue of Coenzyme B12
Source: Angew Chem Int Ed Engl. 2016 Jun 29;55(37):11281–6. doi: 10.1002/anie.201603738 (PMC5103170; doi:10.1002/anie.201603738)
Supplement: Supplementary file 1 — Supplementary [file ANIE-55-11281-s001.pdf]

Angewandte Chemie
Eine Zeitschrift der Gesellschaft Deutscher Chemiker

Supporting Information

Total Synthesis, Structure, and Biological Activity of Adenosylrhodibalamin, the Non-Natural Rhodium Homologue of Coenzyme B_{12}

Florian J. Widner, Andrew D. Lawrence, Evelyne Deery, Dana Heldt, Stefanie Frank, Karl Gruber, Klaus Wurst, Martin J. Warren, and Bernhard Kräutler**

anie_201603738_sm_miscellaneous_information.pdf

Methods

General information

Materials: 5'-Deoxy-5'-iodoadenosine prepared as published [1], (2-amino-1-methylethyl)-3'-(α-ribazolyl)-diphosphate (B_{12}-nucleotide) [2], recrystallized from H_2O / acetonitrile (ACN) at 4°C. Water deionized using Epure, Barnstead Co. Methanol (MeOH) distilled over Mg, acetic acid distilled over P_2O_5, ACN and MeOH, HPLC gradient grade from BDH prolabo, acetone, tertiary-butanol, sodium acetate, puriss. p.a., hydroxybenzotriazole, 1-ethyl-3-(3-dimethylaminopropyl)carbodiimide hydrochloride, potassium dihydrogen phosphate, di-potassium hydrogen phosphate, sodium borohydride, purum, potassium chloride, potassium cyanide 'ultra' were from Fluka, propane-1,2-diol, reagent gr., ammonium chloride, calcium chloride, sodium chloride, sodium carbonate (anhydrous), adenosine triphosphate, ammonium persulfate, chloramphenicol, 4-(2-hydroxyethyl)-1-piperazineethanesulfonic acid (HEPES), imidazole, L-arabinose, L-glutamine, 3-methyl-2-benzothiazolinone hydrazine hydrochloride hydrate (MBTH), ampicillin, puriss., DEAE-sephacel, were from Sigma Aldrich, Sep-Pak-C18 Cartridges, Waters Associates. LiChroprep RP-18 (25-40µm) and TLC aluminium sheets, silica gel 60 RP-18 F_{254}S, Merck.

Instruments: UV/Vis: Hitachi-U3000, λ_{max} (log ε) in nm; or Agilent Cary 60 (kinetic measurements). ^{1}H- and ^{13}C-NMR: Bruker UltraShield 600 MHz or 500 MHz Varian Unity Inova, equipped with 5 mm triple-resonance probe with z-gradients. ESI-MS: Finnigan MAT95-S or Finnigan LCQ classic**,** (+)-ion mode, spray voltage 1.4 kV, m/z (relative intensity in %, signals > 10 %); samples applied to RP18-cartridge, washed with 0.1 % aqueous acetic acid, eluted with MeOH/H_2O (9:1). Hitachi HPLC-system, manual sampler, C18 column (Phenomenex Hyperclone 250 x 4.6 mm, BDH prolabo, LiChroprep RP-18 (25-40µm)), flow: 1.0 ml min^{-1}. LC-MS: Agilent 1100 series HPLC, C18 column (Ace 5 AQ column 150 × 2.1 mm, 5 µm; Advanced Chromatography Technologies), flow: 0.2 ml min^{-1}, coupled to micrOTOF-Q II mass spectrometer (Bruker). Fast protein liquid chromatography, AktaFPLC, gel filtration column (Sephacryl S300 High Resolution, (25 - 75 µm)), flow rate of 1.3 ml min^{-1}.

Strains:

(1) BL21 star(DE3)(pLysS-DNAJ^{RC}-ORF647^{RC}) (pETcoco2-(A)^{Mbar}(IG)^{Bmei}(JFMKLH)^{RC}(B)^{Bmei})

(2) BL21 star(DE3)(pLysS-DNAJ^{RC}-cobB^{RC}) (pET3a-cobB^{Bmei})

(3) BL21 star(DE3)(pET14b-cobQ^{RC})

(4) BL21 star(DE3) (pLysS-His-pduC) (pET3a-pduDE) [3]

^{RC}: *Rhodobacter capsulatus*, ^{Mbar}: *Methanosarcina barkeri*, ^{Bmei}: *Brucella Melitensis*

Construction of plasmids.

All genes were amplified by PCR using genomic DNA from Rhodobacter capsulatus SB1003 (cobJ, cobF, cobM, cobK, cobL, cobH, DNAJ, ORF647 and cobB), Brucella melitensis 16M (cobI, cobG, cobB), Methanosarcina barkeri (cobA) or Citrobacter freundii (pduC, pduD and pduE). All the amplified genes were cloned into a modified pET3a except pduC, which was cloned into pET14b, which allowed its fusion to a 5' histidine tag sequence. Subsequently, the genes were either cloned contiguously within a plasmid or transferred to pLysS. The plasmids pET3a, pET14b and pLysS were originally bought from Novagen/Merck Millipore, UK. The final plasmids are described in the Strains section.

Preparation of hydrogenobyrinic acid a,c-diamide (Hbad) [4]:

Following plasmid transformation in *E. coli* strain BL21 star(DE3) (see strain 1) the cells were streaked on an LB agar plate and incubated overnight at 28° C with 100 µg/ml ampicillin and 34µg/ml chloramphenicol for selection. The 10 ml LB starter cultures were inoculated with a single colony and incubated under shaking (160 rpm) overnight at 28 °C. The 1 L cultures were prepared containing 10 g yeast extract, 16 g tryptone, 5 g NaCl, 1 g NH_4Cl and inoculated by adding pre-cultures and incubated under shaking (160 rpm) at 28 °C for 6 hours. L-arabinose was added at 0.2% (w/v) and the cultures were incubated overnight under shaking (160 rpm) at 28 °C. After centrifugation the pellets from 50 L of culture, grown under these conditions, were re-suspended in 400 ml of hepes buffer pH 7.5 (10 mM). The red brown coloured suspension was sonicated, centrifuged and the dark red supernatant was boiled for 5 min and centrifuged. The orange product was purified by DEAE column chromatography and desalted using a SepPak RP18 column, yielding 121 µmol of a hydrogenobyrinic acid (**Hba**)/**Hbad** mixture (photometrically determined, ratio 60/40).

After plasmid transformation in *E. coli* strain BL21 star(DE3) (see strain 2) the recombinant strain was grown aerobically in LB at 37° C with 100 µg/ml ampicillin and 34µg/ml chloramphenicol for selection until they reached an OD_{600} of 0.6. The cells were incubated overnight at 18° C after adding IPTG to a final conc. of 0.4 mM for protein overproduction. After centrifugation the pellets of 14 liters of culture were re-suspended in 160 ml of 20 mM Tris HCl buffer pH 8.0 containing 100 mM NaCl, lysed by sonication and added to the pH 7 buffered previously prepared aq. mixture of Hba/Hbad (121 µmol) containing 1.5 g ATP, 0.57 g $MgCl_2$ and 0.44 g L-glutamine. The reaction solution was kept at room temperature in the dark overnight. The red suspension was boiled for 5 min and centrifuged. The supernatant was purified by DEAE column chromatography as well as reversed phase chromatography (30 g RP-18, orange Hbad was eluted with 1 % aq. acetic acid / 50 % ethanol) and desalted on a

Sep-Pak column. The solvent was removed using a rotary evaporator. The orange residue was re-dissolved in water and lyophilized yielding 88.2 mg **Hbad** (100.3 µmol).

Dichlororhodibyrinic acid a,c-diamide (DCRhbad)

In a 25 ml two necked round bottom flask, 10.0 mg of **Hbad** (11.4 µmol) and 23 mg anhydrous sodium acetate (280 µmol) were dissolved in 2 ml acetic acid and degassed with argon in the dark for 10 min. A yellow solution of 25.5 mg rhodium(I) dicarbonyl chloride dimer (65.6 µmol) dissolved in 5 ml tert-butanol was added. The mixture was stirred at room temperature in the dark for 24 hours. The orange brown solution was diluted with saturated aqueous sodium chloride solution and methanol was evaporated on a rotary evaporator. The remaining aqueous solution was applied to a 1 g RP-18 cartridge, washed with saturated aqueous sodium chloride solution (15 ml) followed by elution of an orange substance with MeOH / 1 M aqueous sodium chloride solution (9:1, 15 ml). The solution of raw DCRhbad was directly used for alkylation to adenosylrhodibyrinic acid-a,c-diamide (AdoRhbad) (see below). For quantification, an equivalent of DCRhbad was converted into dicyano-rhodibyrinic acid-a,c-diamide ($(CN)_2$Rhbad) by adding KCN. Yield: 9.7 mg **DCRhbad** (9.2 µmol, 81 %).

UV/Vis: (MeOH / 1M aq. NaCl, approx. c = 0.7 x 10^{-4} M): λ_{max}(rel. ε) = 512 (0.39) nm, 486(0.42), 408(0.21), 389(0.22), 343(1.00), 289(0.72), 272(0.83); **ESI-MS**: m/z(%) = 1018.33(15), 1017.40(32); 1016.31(49), 1015.40(100, $[M-Cl]^+$); $C_{45}H_{61}ClN_6O_{12}Rh^+$: m/$z_{calc}$(%): 1018.31(16), 1017.31(36), 1016.31(51), 1015.31(100).

Adenosylrhodibyrinic acid a,c-diamide (AdoRhbad)

In a UV/Vis-cuvette, a solution of DCRhbad (0,86 µmol) in 1 ml MeOH / aq. sodium chloride (1 M) (9:1) was degassed with argon for 15 min. The mixture was cooled with an ice bath, roughly 100 eq. of sodium borohydride were added and the cuvette was closed with a Teflon stopper. After 30 minutes the UV/Vis spectrum showed the spectrum of a reduced rhodium(I)-corrin [6]). 5'-Deoxy-5'-iodoadenosine (2.4 mg, 8.6 µmol, 10 eq.) was added to the light yellow solution. All further operations were carried out in a dark room. The solution was stirred for 4 h in the ice bath followed by 10 hours at room temperature. The resulting cloudy yellow solution was treated with 100 µl acetic acid and centrifuged. The yellow supernatant was diluted with 2 ml distilled water. MeOH was removed with a rotary evaporator. The remaining aqueous solution was desalted using a 1 g RP-18 cartridge and dried on a rotary evaporator. The crude product was purified by preparative HPLC, desalted using a RP-18 cartridge and dried on a rotary evaporator. The residue was dissolved in 0.3 ml of water and lyophilized yielding 1.0 mg **AdoRhbad** (0.80 µmol, 75 %).

UV/Vis: (H_2O, c = 4.0 x 10^{-5} M): λ_{max} (log ε) = 487 (sh, 4.08) nm, 469 (4.11), 416 (3.78), 398 (3.70), 340 (4.45), 289 (sh, 4.26) 269 (4.46), 255 (4.47) nm; **CD:** (H_2O, c = 4.0 x 10^{-5} M): λ_{max} ($\Delta\varepsilon$), λ_{min} ($\Delta\varepsilon$) = 488 (-0.8), 393 (0.3), 346 (1.3), 308 (-0.4), 280 (-0.3), 263 (0.3), nm (l mol^{-1} cm^{-1}); λ_0: 532, 440, 326, 272, 255 nm; **ESI-MS**: m/z (%) = 1232.34 (18), 1231.26 (52), 1230.27 (68, $[M+H]^+$); 1096.26 (18), 1095.17 (32, $[M+H\text{-}Ade]^+$); 980.20 (10, $[M+H\text{-}5'Ado]^+$); 616.52 (20), 616.03 (65) 615.57 (100, $[M+2H]^{2+}$); 490.50 (15), 489.96 (18, $[M+2H\text{-}5'Ado]^{2+}$); $C_{55}H_{73}N_{11}O_{15}Rh^+$: m/$z_{calc}$ (%) = 1232.44 (21), 1231.44 (61), 1230.43 (100);

^{1}H-NMR: (600 MHz, 298 K, D_2O, 10 mM potassium phosphate pD 7.4, c = 1.6 x 10^{-3} M): δ = -0.54 (app. *t, J* = 10.5 Hz, 1H, HC-5RLβ (pro-R C-5RL)), 0.18 (app. *d, J* = 11.8 Hz, 1H, HC-5RLα (pro-S C-5RL)), 0.93 (*s*, 3H, H_3C-12B), 1.28 (*s*, 3H, H_3C-1A), 1.37 (*s*, 3H, H_3C-17B), 1.56 (*s*, 6H, H_3C-2A, H_3C-12A), 1.73 (*s*, 3H, H_3C-7A), 1.75 (*m*, 1H, HC-81), 1.92 (*m*, 1H, HC-171), 2.01 (*m*, 1H, HC-131), 2.02 (*m*, 1H, HC-171), 2.10 (*m*, 1H, HC-4RL), 2.11 (*m*, 1H, HC-31), 2.14 (*m*, 2H, H_2C-172), 2.18 (*m*, 1H, HC-81), 2.21 (*m*, 2H, H_2C-71), 2.24 (*m*, 1H, HC-131), 2.32 (*m*, 1H, HC-31), 2.46-2.52 (*m*, 6H, H_2C-21, H_2C-82, H_2C-132 superimposed by 2.43 (*s*, 3H, H_3C-51), 2.49 (*s*, 3H, H_3C-151)), 2.64 (m, 2H, H_2C-32), 2,73 (*m*, 2H, H_2C-181), 3.03 (*m*, 1H, HC-18), 3.36 (*dd*, *J* = 2.6, 6.4 Hz, 1H, HC-13), 3.41 (app. *t, J* = 6.0 Hz, 1H, HC-3RL), 3.78 (*dd,* J = 4.7, 8.7 Hz, 1H, HC-8), 4.11 (*d*, *J* = 9.0 Hz, 1H, HC-3), 4.29 (*d*, *J* = 10.8 Hz, 1H, HC-19), 4.31 (dd, J = 3.4, 5.5 Hz, 1H, HC-2RL), 5.48 (*d*, *J* = 3.4 Hz, 1H, HC-1RL), 6.43 (*s,* 1H, HC-10), 7.96 (*s,* 1H, HC-8L), 8.25 (*s,* 1H, HC-2L);

Adenosylrhodibyric acid (AdoRhby)

Recombinant expression of CobQ in *E. coli*: After plasmid transformation in *E. coli* strain BL21 star(DE3) (see strain 3) the recombinant strain was grown aerobically in LB at 37° C for 6 hours with 100 µg/ml ampicillin for selection. The protein production was induced by adding 400 µl of IPTG (1M) and incubated overnight under shaking (160 rpm) at 19 °C. After centrifugation the pellet was resuspended with 30 ml tris buffer pH 8 (20 mM) containing sodium chloride (500mM) and imidazole (10mM), sonicated, centrifuged and the supernatant was applied to a Ni^{2+}-column. The enzyme eluted at 400 mM imidazole. The enzyme solution was immediately buffer exchanged (ches pH 9.0 (20 mM) containing sodium chloride (100 mM)) using a PD10 column. The solution was concentrated by using VIVA-spin tubes to a protein concentration of 3.3 mg/ml (58 µM).

Enzymatic synthesis of AdoRhby: All further operations were carried out in a dark room. In a 10 ml round bottom flask 2.4 mg AdoRhbad (1.9 µmol) were dissolved in 840 µl H_2O and 300 µl aqueous ATP solution (100 mM, pH 7), 60 µl aqueous $MgCl_2$ solution (1 M), 150 µl aqueous L-glutamine solution (100 mM) and 1.5 ml Cob Q solution (58 µM) were added. After

incubation at room temperature overnight the reaction was stopped by adding 300 µl acetic acid, centrifuged and the supernatant was purified by preparative HPLC. The product fraction was desalted using a RP-18 cartridge and dried on a rotary evaporator. The residue was dissolved in 0.5 ml of H_2O and lyophilized yielding 2.2 mg **AdoRhby** (1.8 µmol, 92 %).

UV/Vis: (H_2O, c = 4.0 x 10^{-5} M): λ_{max} (rel. ε) = 486 (sh, 0.39) nm, 469 (0.40), 416 (0.22), 398 (0.18), 342 (0.90), 290 (sh, 0.64), 265 (0.92), 256 (100) nm;

^{1}H-NMR: (600 MHz, 298 K, D_2O / aqueous potassium phosphate pH 5.0 (10 mM) (1:9), c = 0.5 x 10^{-3} M): δ = -0.60 (*m,* 1H, HC-5RLβ), 0.14 (apparent *d, J* = 11.9 Hz, 1H, HC-5RLα), 0.87 (*s*, 3H, H_3C-12B), 1.28 (*s*, 3H, H_3C-1A), 1.37 (*s*, 3H, H_3C-17B), 1.56 (*s*, 3H, H_3C-2A), 1.57 (*s*, 3H, H_3C-12A), 1.72 (s, 3H, H_3C-7A), 2.40 (*s*, 3H, H_3C-51), 2.46 (*s*, 3H, H_3C-151), superimposed by 1.74-2.47 (m, total ca. 31 H), 2.48-2.63 (m, 2H, H_2C-32 ?), 2,75 (*m*, 2H, H_2C-181 ?), 3.03 (*m*, 1H, HC-18), 3.26 (*m*, 1H, HC-13), 3.39 (apparent *t, J* = 6.3 Hz, 1H, HC-3RL), 3.76 (*dd,* J = 5.0, 8.1 Hz, 1H, HC-8), 4.05 (apparent *d*, *J* = 8.8 Hz, 1H, HC-3), 4.26 (*d*, *J* = 10.9 Hz, 1H, HC-19), 4.32 (dd, J = 3.6, 5.6 Hz, 1H, HC-2RL), 5.45 (*d*, *J* = 3.4 Hz, 1H, HC-1RL), 6.40 (*s,* 1H, HC-10), 7.89 (*s,* 1H, HC-8L), superimposed by 6.70-7.95 (m, 12 amides), 8.20 (*s,* 1H, HC-2L);

HPLC/ESI-MS: (RP18 Ace 5 AQ column, 150 x 2.1 mm, 5 µm, flow 0.2 ml min^{-1}, 0.1 % aqueous TFA, ACN, linear gradient 0 – 100 % ACN in 40 min, online UV/Vis detection at 469 nm): m/z (%) = 1226.48 (5, $[M+H]^+$), 625.76 (15), 625.26 (72), 624.75 (100, $[M+H+Na]^{2+}$);

Adenosyl-rhodibalamin (AdoRbl)

All operations were carried out in the dark. In a small glass tube 0.30 mg AdoRhby (0.24 µmol), 0.30 mg B_{12}-nucleotide group (0.72 µmol, 3 eq) and 0.47 mg hydroxybenzotriazol (3.5 µmol) were dissolved in 150 µl H_2O and cooled to 0 °C. A solution of 1-ethyl-3-(3-dimethylaminopropyl)carbodiimide hydrochloride (0.78 mg, 4.1 µmol) in 50 µl ice-cold H_2O was added and the mixture was stirred for 2 hours at 0°C and additional 20 hours at room temperature. The crude product was purified by preparative HPLC, desalted using a 1 g RP-18 cartridge. The solvent was evaporated on a rotary evaporator. The orange residue was dissolved in 0.1 ml of H_2O and lyophilized yielding 0.31 mg **AdoRbl** (0.19 µmol, 79 %).

UV/Vis: (H_2O, c = 1.4 x 10^{-5} M): λ_{max} (log ε) = 512 (4.06) nm, 491 (4.06), 424 (3.69), 403 (3.72), 350 (4.49), 289 (sh, 4.44), 259 (4.66) nm;

^{1}H-NMR: (500 MHz, 298 K, c = 0.4 x 10^{-3} M in 10 mM potassium phosphate, D_2O, pD 7.4): δ = -0.74 (apparent *t, J* = 9.5 Hz, 1H, HC-5RLβ (pro-R C-5RL)), 0.24 (apparent *d, J* = 11.8 Hz, 1H, HC-5RLα (pro-S C-5RL)), 0.70 (*s*, 3H, H_3C-1A), 0.89 (m, 1H, HC-81), 0.92 (*s*, 3H, H_3C-12B), 1.00 (m, 1H, HC-82), 1.23 (d, *J* = 6.2 Hz, 3H, H_3C-177), 1.40 (m, 1H, HC-81 superim-

posed by 1.41 (*s*, 3H, H_3C-12A)), 1.49 (*s*, 3H, H_3C-17B), 1.50 (*s*, 3H, H_3C-2A), 1.60 (m, 1H, HC-82), 1.61 (m, 1H, HC-172), 1.79 (s, 3H, H_3C-7A), 1.85 (m, 1H, HC-31), 1.98 (m, 1H, HC-131), 2.04 (m, 1H, HC-131), 2.14-2.27 (*m*, 2H, HC-31, HC-171 superimposed by 2.21 (*s*, 3H, H_3C-11N), 2.24 (*s*, 3H, H_3C-10N)), 2.38-2.50 (m, 4H, HC-21, H_2C-71, HC-4RL, HC-171), 2.55-2.64 (*m*, 6H, HC-21, H_2C-32, H_2C-132, HC-171 superimposed by 2.57 (*s*, 3H, H_3C-151), 2.60 (*s*, 3H, H_3C-51)), 2.72 (*dd*, *J* = 3.0, 15.9 Hz, 1H, HC-181), 2,82 (*m*, 1H, HC-181), 2.86 (*m*, 1H, HC-18), 3.00 (apparent *d*, *J* = 10.2 Hz, 1H, HC-13), 3.17 (*m,* 1H, HC-175), 3.38 (*t, J* = 6.0 Hz, 1H, HC-3RL), 3.56 (*m,* 1H, HC-175), 3.58 (*m,* 1H, HC-8), 3,74 (*m,* 1H, HC-5R), 3,89 (*m,* 1H, HC-5R), 4.08 (*m,* 1H, HC-4R), 4.16 (*d*, *J* = 7.8 Hz, 1H, HC-3), 4.20 (*m,* 1H, HC-2R), 4.23 (*d*, *J* = 10.9 Hz, 1H, HC-19), 4.36 (*m,* 1H, HC-176), 4.47 (dd, J = 3.8, 5.0 Hz, 1H, HC-2RL), 4.70 (*m,* 1H, HC-3R), 5.51 (*d*, *J* = 3.3 Hz, 1H, HC-1RL), 6.03 (*s,* 1H, HC-10), 6.18 (*s,* 1H, HC-4N), 6.19 (*d*, *J* = 2.7 Hz, 1H, HC-1R), 6.61 (*s,* 1H, HC-2N), 7.12 (*s,* 1H, HC-7N), 7.95 (*s,* 1H, HC-8L), 8.25 (*s,* 1H, HC-2L).

ESI-MS: (H_2O/MeOH): m/z (%) = 1663.53 (51), 1662.60 (84), 1661.67 (86, $[M+K]^+$); 1647.53 (53), 1645.53 (92), 1645.53 (100, $[M+Na]^+$); 1285.67 (15), 1284.73 (28), 1283.73 (33, $[M-C_{14}H_{16}N_2O_6P]^+$); 835.33 (13), 834.87 (24), 834.40 (15, $[M+2Na]^{2+}$); $C_{72}H_{100}N_{18}NaO_{17}PRh^+$: m/z_{calc} (%) = 1647.63 (31), 1646.62 (85), 1645.62 (100).

X-ray crystallography

Crystals of **AdoRbl** were grown from H_2O/ACN. Diffraction experiments were carried out with a Nonius Kapp CCD diffractometer at 233 K. Diffraction data extended to a resolution of 0.71 Å. The asymmetric unit of the orthorhombic crystal contained one molecule of **AdoRbl**, 14 well-ordered H_2O molecules and 2.5 ACN molecules. Indexing of diffraction images, intensity integration, and data scaling were performed with programs DENZO and SCALEPACK.[5] The crystal was monoclinic (space group C2 no. 5) with unit cell constants a=32.9552(2) Å, b=20.8560(3) Å, and c=14.6559(3) Å; β= 108.833(1)°.

The structure was solved by direct methods and refined against F^2-values using the program SHELXL-97.[6] Full matrix least-squares anisotropic refinement converged at R1 = 0.0436 for all data. No absorption correction was applied to the data. The solvent region was modeled using H_2O and ACN molecules with anisotropic atomic displacement parameters (adp). H-Atom positions of **AdoRbl** were calculated and refined as ‘riding’ on their respective non-H-atom. For methyl- and hydroxyl-groups the torsion angle around the C-C or C-O bond was also refined (omitted for water molecules). The isotropic adp for each H-atom was set to 1.5 times (for methyl- and hydroxyl-groups) and 1.2 times (for all other hydrogen atoms) the equivalent isotropic atomic displacement parameters of the adjacent non-H-atom.

Experiments on the *in vitro* characterization of AdoRbl

Recombinant expression of $PduC^{H}DE$ in *E. coli*

After plasmid double transformation in *E. coli* strain BL21 star(DE3) (see strain 4) the recombinant strain was grown aerobically in LB containing 0.1 % propane-1,2-diol at 37° C for 6 hours with 100 µg/ml ampicillin and 34µg/ml chloramphenicol for selection. The protein production was induced by adding 400 µl of IPTG (1M) and incubated overnight under shaking (160 rpm) at 19 °C. After centrifugation the pellet was re-suspended with 30 ml phosphate buffer pH 8 (50 mM) containing sodium chloride (500 mM), imidazole (10 mM) and 2 % propane-1,2-diol, sonicated and centrifuged. All enzyme complex parts were purified by Ni^{2+}-column chromatography and immediately buffer exchanged (phosphate buffer pH 8 (50 mM) containing 2 % propane-1,2-diol) using a Pd10 column. The enzyme complex was purified by gel filtration chromatography using a Sephacryl column S300 and phosphate buffer pH 8 (50 mM) containing 2 % propane-1,2-diol. The enzyme complex containing fractions were concentrated using VIVA-spin to 9.3 µg/ml $PduC^{H}DE$.

In vitro activity and competitive inhibition assays

All operations were carried out in a dark room. The activity of the diol dehydratase was determined by the 3-methyl-2-benzothiazolinone hydrazone (MBTH) method.[7] Experiments were done in duplicates (0, 0.25, 0.5, 1.0, 1.5, 2.0, 2.5, 3.5, 5, 10, 15, 30 µM **AdoCbl**) with 0.2 µg $PduC^{H}DE$ in each assay. Kinetic data of $PduC^{H}DE$ with **AdoCbl**: K_m = 3.0 µM; V_{max} = 103 µmol/min/mg) **AdoRbl** had no activity with the **AdoCbl** dependent diol dehydratase $PduC^{H}DE$.

For the inhibition assays the activity of the diol dehydratase was determined by adding 0, 2, 4, 6, 10, 20 µM of **AdoRbl** or 0, 0.2, 1, 2, 4, 20 µM vitamin B_{12} to the **AdoCbl** activity assay. Thereby, apparent K_m ($K_m$app) and V_{max} values were obtained by solving the Michaelis-Menten equation which led to the inhibitory effect (K_i value) [8]. K_i(**AdoRbl**) = 6.9 µM (±0.3 µM), K_i(vitamin B_{12}) = 2.5 µM (±0.2 µM)

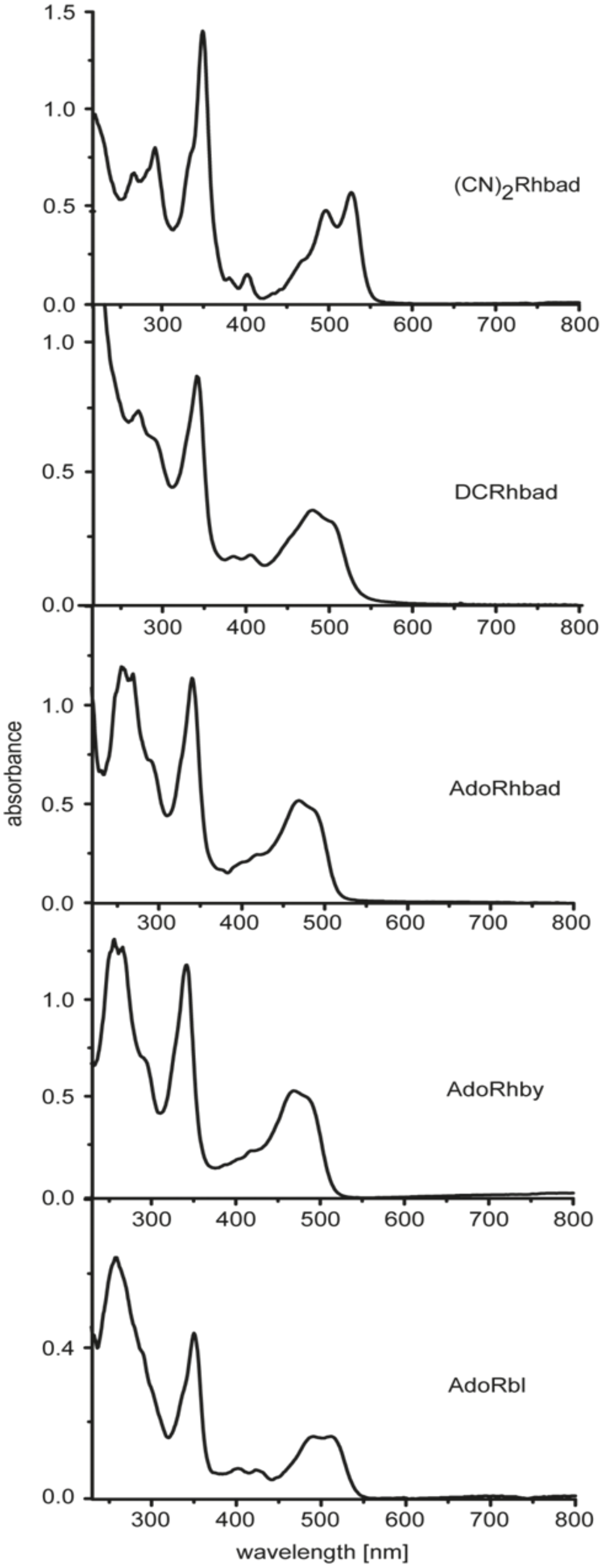

Figure S1. UV/Vis spectra of rhodium corrins. Dicyanorhodibyrinic acid a,c-diamide (**(CN)$_2$Rhbad**, c = 4.2 x 10^{-5} M, 0.1 % aqueous HCN), dichlorohodibyrinic acid a,c-diamide (**DCRhbad**, c = 7.0 x 10^{-5} M, MeOH / 1M aqueous NaCl solution), adenoslyrhodibyrinic acid a,c-diamide (**AdoRhbad**, c = 4.0 x 10^{-5} M, H_2O), adenoslyrhodibyric acid (**AdoRhby**, c = 4.0 x 10^{-5} M, H_2O), adenosylrhodibalamin (**AdoRbl**, c = 1.4 x 10^{-5} M, H_2O)

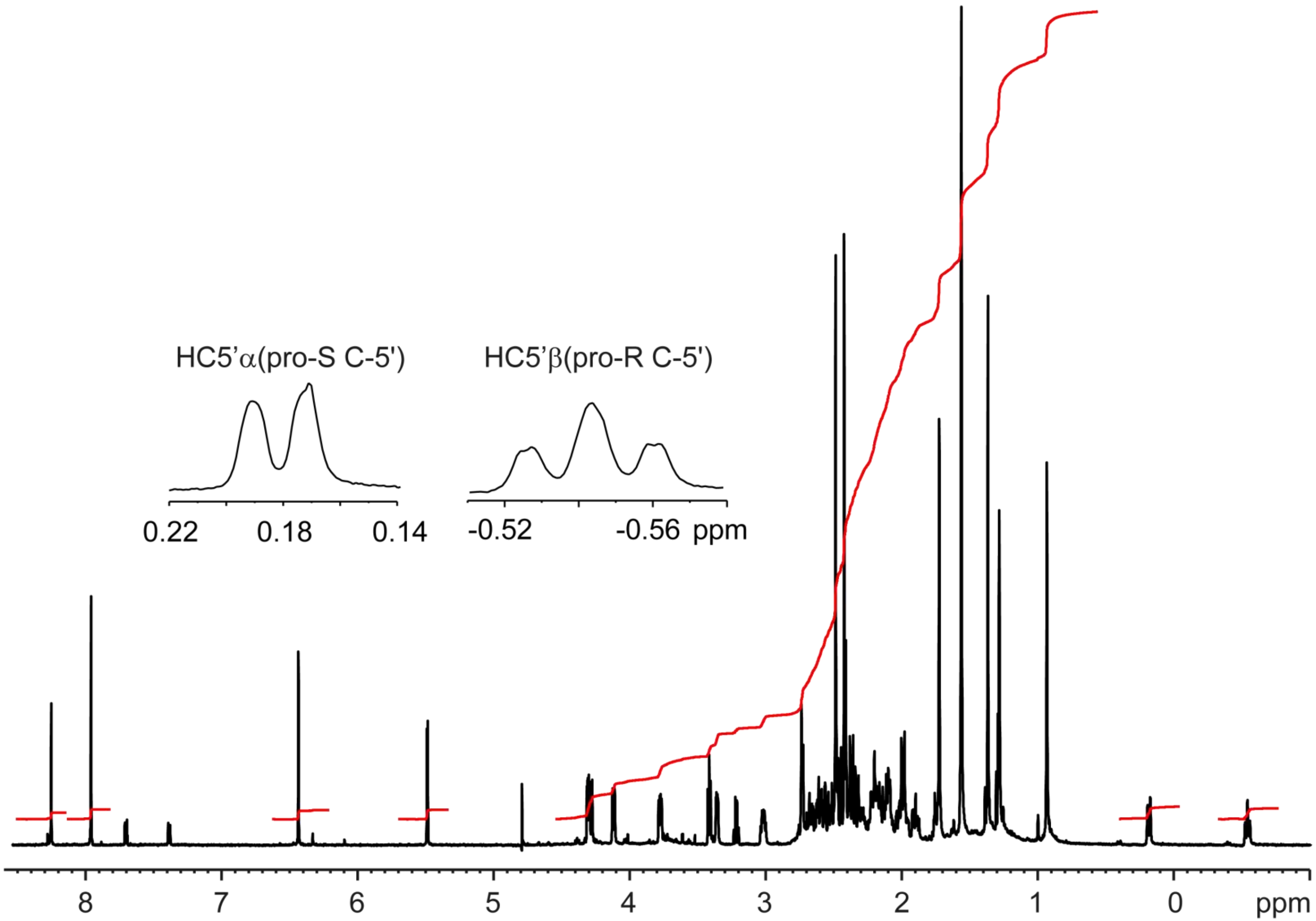

Figure S2. 600 MHz ^{1}H-NMR spectrum of adenosylrhodibyrinic acid a,c-diamide (**AdoRhbad**, c = 1.6 x 10^{-3} M, D_2O, 10 mM potassium phosphate pD 7.4, 298 K, suppression of HDO-signal); inset shows enlarged section of the spectrum and 1.7 Hz Rh-coupling of protons of Rh-bound H_2-C5.

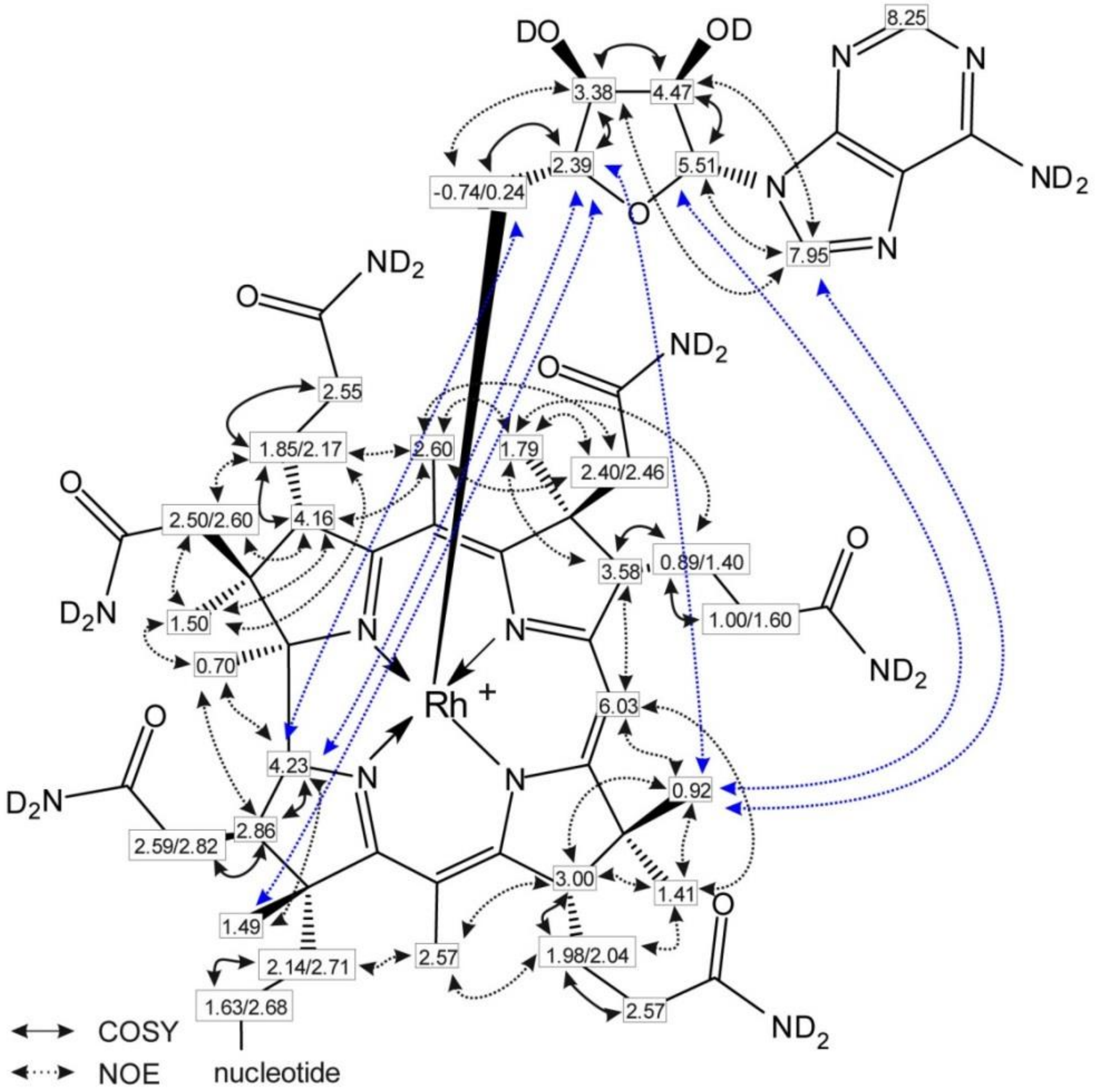

Figure S3. ^{1}H NMR signal assignment of the corrin ligand and adenosyl moiety of adenosyl-rhodibalamin (**AdoRbl**) and 2D homonuclear correlations from 500 MHz ^{1}H NMR spectra (298 K, D_2O, 10 mM potassium phosphate pD 7.4). Blue dashed lines signify NOE correlations between adenosyl moiety and the corrin ring; black lines: correlations within corrin moiety from NOE- (dashed lines) and COSY-couplings (full lines).

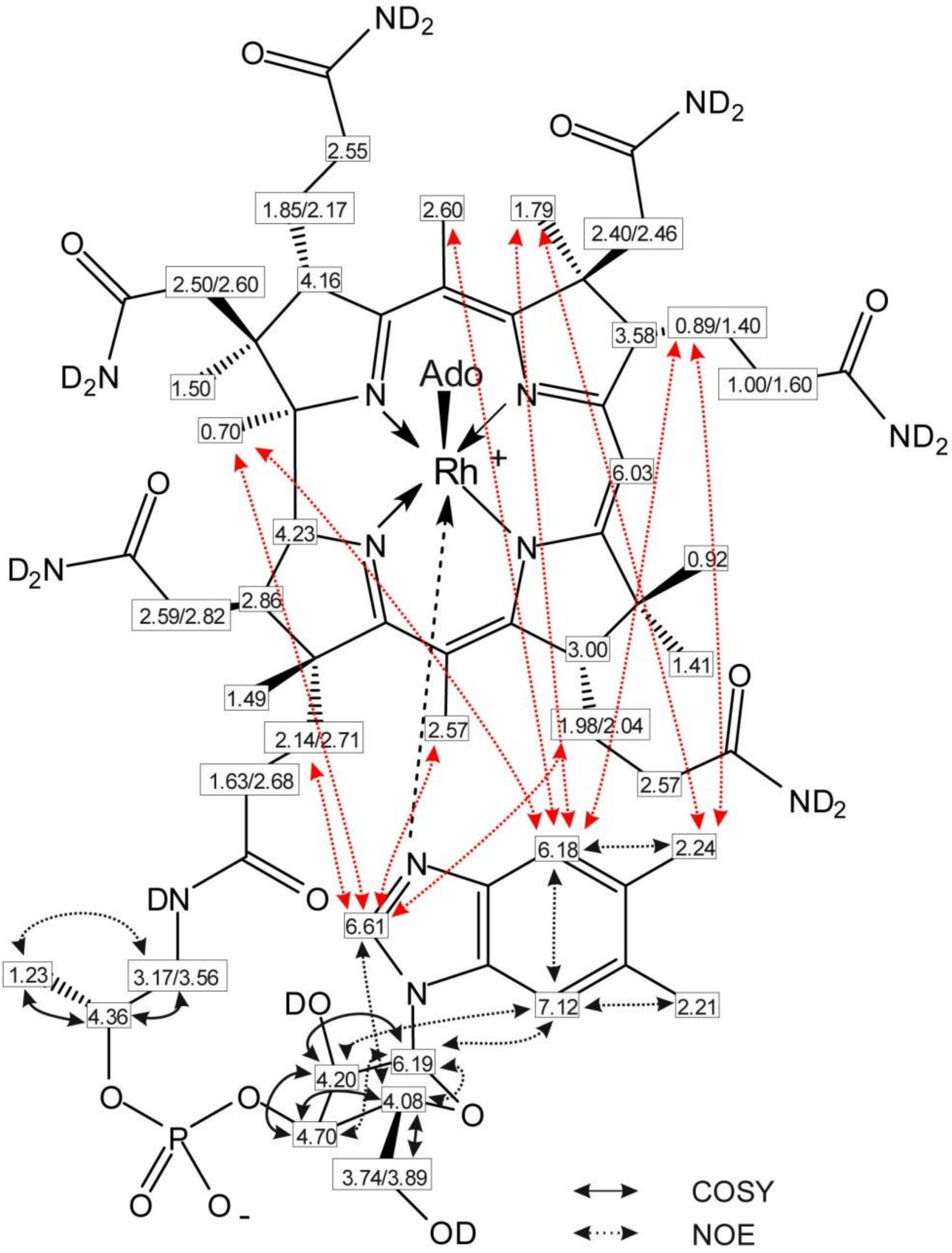

Figure S4. ^{1}H NMR signal assignment of the nucleotide loop of adenosylrhodibalamin (**AdoRbl**) and 2D homonuclear correlations from 500 MHz ^{1}H NMR spectra (298 K, D_2O, 10 mM potassium phosphate pD 7.4). Blue dashed lines signify NOE correlations between dimethyl benzimidazole moiety and the corrin ring; black lines: correlations within nucleotide loop moiety from NOE- (dashed lines) and COSY-couplings (full lines).

Table S1. Chemical-shift data (δ in ppm) in ^{1}H- and ^{13}C-NMR spectra and signal assignments of corrin ring part of **AdoRbl** in comparison with coenzyme B_{12} (**AdoCbl**). See Fig. S5 for numbering.

	^{1}H (**AdoRbl**)	^{13}C (**AdoRbl**)	^{1}H (**AdoCbl**)	^{13}C (**AdoCbl**)
C1		88.6		88.5
C1A	0.70	23.8	0.47	23.5
C2		50.3		49.5
C2A	1.50	19.4	1.36	19.9
C21	2.50 / 2.60	45.6	2.41	46.2
C22		179.3		179.3
C3	4.16	58.1	4.10	58.5
C31	1.85 / 2.17	29.0	1.96 / 2.06	29.2
C32	2.55	38.0	2.50	38.3
C33		180.7		181.1
C4		173.3		178.7
C5		109.1		108.4
C51	2.60	18.6	2.45	18.3
C6		165.3		166.6
C7		53.9		53.1
C7A	1.79	21.2	1.70	21.7
C71	2.40 / 2.46	45.6	1.72 / 2.19	45.3
C72		179.6		177.9
C8	3.58	57.1	3.29	57.5
C81	0.89 / 1.86	26.3	0.81 / 1.75	28.7
C82	1.00 / 1.61	34.6	0.88 / 1.73	34.8
C83		177.9		180.3
C9		169.1		173.1
C10	6.03	95.8	5.93	97.7
C11		172.3		177.6
C12		49.7		49.5
C12A	1.41	23.8	0.32	23.9
C12B	0.92	33.8	0.87	34.2
C13	3.00	56.5	2.89	55.8
C131	1.98 / 2.04	27.7	2.00 / 2.22	30.3
C132	2.57	38.0	2.54	38.1
C133		178.0		181.1
C14		165.1		167.2
C15		108.0		106.9
C151	2.57	19.1	2.43	18.8
C16		173.5		178.7
C17		60.5		60.8
C17B	1.49	19.4	1.36	19.6
C171	2.50 / 2.59	34.2	2.06 / 2.45	34.4
C172	1.60	34.2	1.78	34.6
C173		175.3		178.2
C18	2.86	43.0	2.65	42.5
C181	2.72 / 2.82	34.4	2.65	34.8
C182		179.6		179.0
C19	4.23	77.2	4.24	76.8

Table S2. Chemical-shift data (δ in ppm) in ^{1}H- and ^{13}C-NMR spectra and signal assignments of nucleotide part of **AdoRbl** in comparison with coenzyme B_{12} (**AdoCbl**). See Fig. S5 for numbering.

	^{1}H (**AdoRbl**)	^{13}C(**AdoRbl**)	^{1}H (**AdoCbl**)	^{13}C (**AdoCbl**)
C175	3.17 / 3.56	47.3	3.16 / 3.54	47.8
C176	4.36	75.5	4.33	76.0
C177	1.23	21.2	1.21	21.7
C1R	6.19	89.0	6.26	89.4
C2R	4.20	71.6	4.23	72.0
C3R	4.70	75.6	4.72	76.2
C4R	4.08	84.0	4.10	84.6
C5R	3.74 / 3.89	62.8	3.74 / 3.88	63.4
C2N	6.61	143.7	6.95	144.7
C4N	6.18	120.4	6.24	121.4
C5N		134.7		134.5
C6N		136.7		136.8
C7N	7.12	113.0	7.16	113.5
C8N		132.7		133.3
C9N		139.5		141.0
C10N	2.24	22.3	2.19	22.5
C11N	2.21	21.9	2.19	22.3

Table S3. Chemical-shift data (δ in ppm) in ^{1}H- and ^{13}C-NMR spectra and signal assignments of adeosyl group of **AdoRbl** in comparison with coenzyme B_{12} (**AdoCbl**). See Fig. S5 for numbering.

	^{1}H (**AdoRbl**)	^{13}C(**AdoRbl**)	^{1}H (**AdoCbl**)	^{13}C (**AdoCbl**)
C2L	8.25	155.7	8.19	156.0
C4L		151.7		151.8
C5L		121.7		121.8
C6L		165.5		158.7
C8L	7.95	143.6	8.00	143.8
C1RL	5.51	90.5	5.56	91.0
C2RL	4.47	75.3	4.54	75.6
C3RL	3.38	77.1	3.74	76.6
C4RL	2.39	86.7	2.54	88.6
C5RL	-0.74 / 0.24	23.8	0.57 / 1.55	27.3

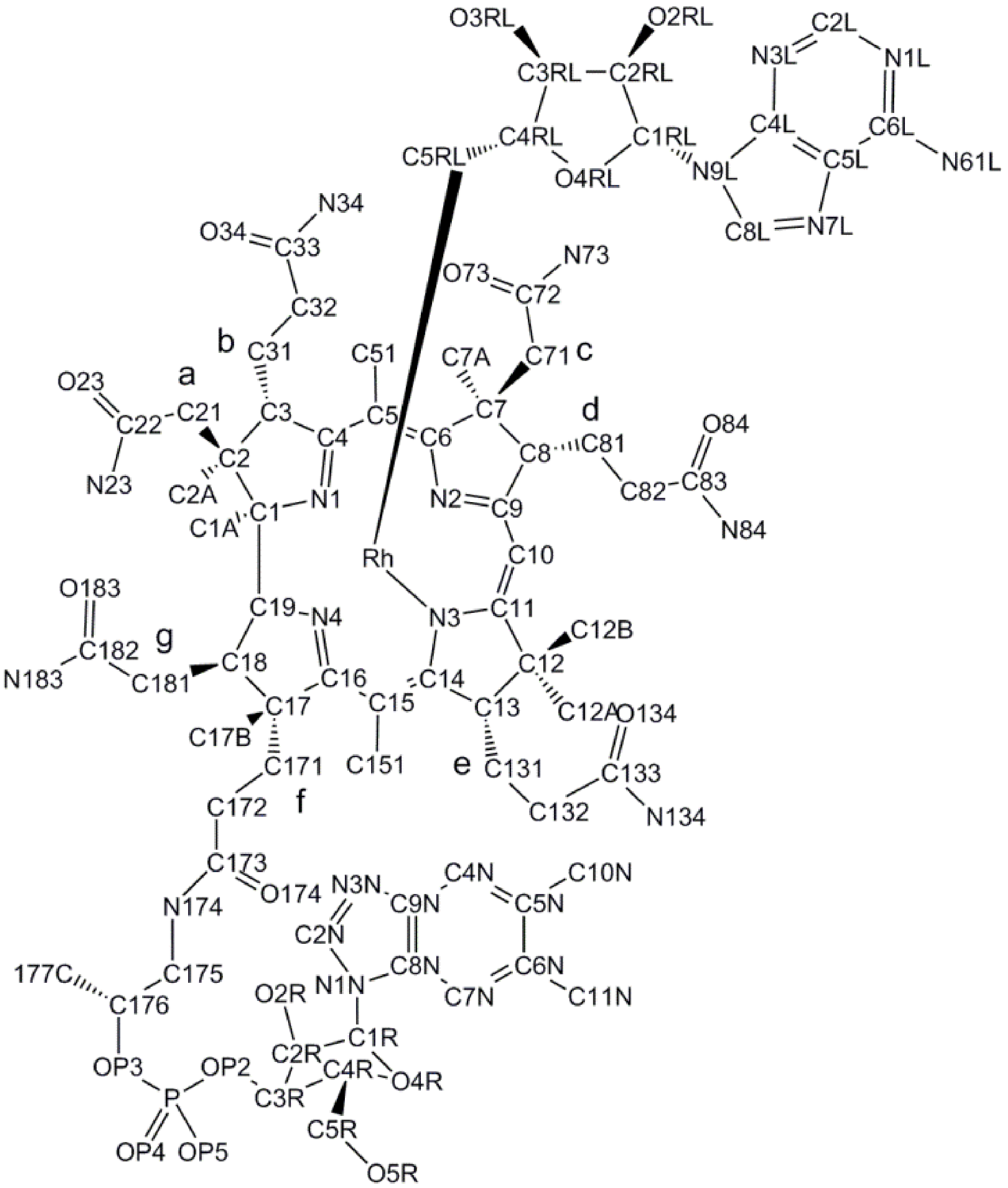

Figure S5. Atom numbering for the rhodium analogue of coenzyme B_{12}, **AdoRbl**, and of other Rh-corrins; acetamide and propionamide side chains are also labelled as “a” to “g” in a clock-wise sense.

Table S4. Crystallographic data for adenosylrhodibalamin (**AdoRbl**)

Empirical formula	C_{72} H_{100} N_{18} O_{17} P Rh x 14 H_2O x 2.5 CH_3CN	
Formula weight	1978.44	
Temperature	233(2) K	
Wavelength	0.71073 Å	
Crystal system	Monoclinic	
Space group	C2 (no. 5)	
Unit cell dimensions	a = 32.9552(2) Å	α= 90°.
	b = 20.8560(3) Å	β= 108.833(1)°.
	c = 14.6559(3) Å	γ = 90°.
Volume	9533.9(2) $Å^3$	
Z	4	
Density (calculated)	1.378 Mg/m^3	
Absorption coefficient	0.284 mm^{-1}	
F(000)	4196	
Crystal size	0.42 x 0.25 x 0.12 mm^3	
Theta range for data collection	1.95 to 25.00°.	
Index ranges	-34<=h<=39, -24<=k<=23, -17<=l<=17	
Reflections collected	26901	
Independent reflections	16360 [R(int) = 0.0210]	
Reflections [I>2sigma(I)]	15391	
Completeness to theta = 25.00°	99.8 %	
Absorption correction	None	
Refinement method	Full-matrix least-squares on F^2	
Data / restraints / parameters	16360 / 1 / 1220	
Goodness-of-fit on F^2	1.060	
Final R indices [I>2sigma(I)]	R1 = 0.0394, wR2 = 0.1008	
R indices (all data)	R1 = 0.0436, wR2 = 0.1036	
Absolute structure parameter	-0.016(15)	
Largest diff. peak and hole	0.756 and -0.513 e.$Å^{-3}$	

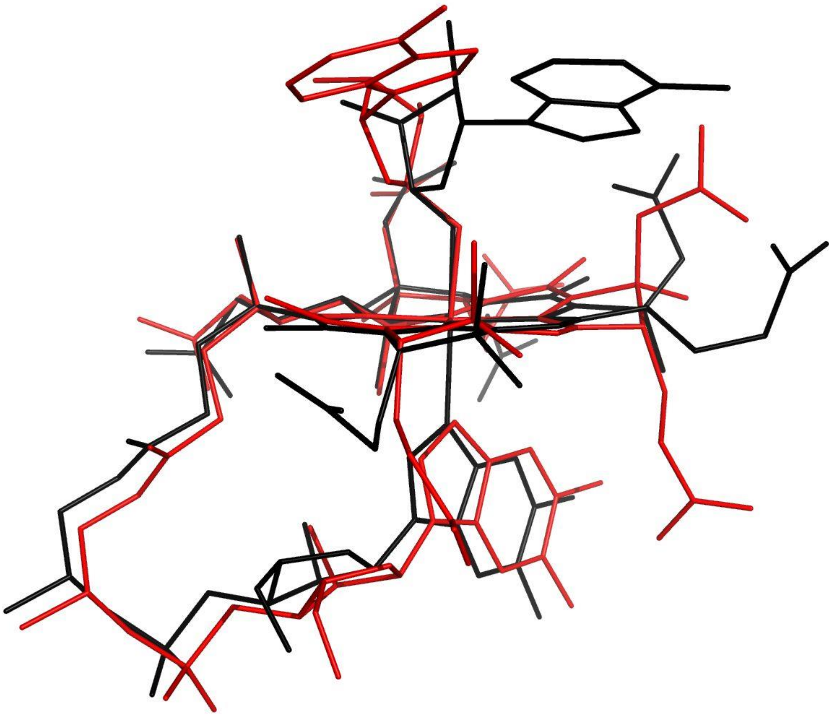

Figure S6. Superposition of crystal structures of adenosylrhodibalamin (**AdoRbl**, black trace) and of coenzyme B_{12} (**AdoCbl**, red trace), using 'stick-models'.

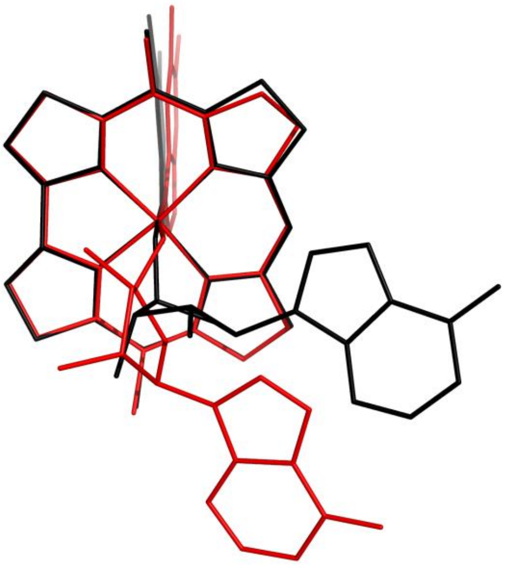

Figure S7. Superposition of core moieties of **AdoRbl** (black) and **AdoCbl** (red trace) in 'stick-models', highlighting the counter-clockwise rotation of the upper adenosyl ligand.

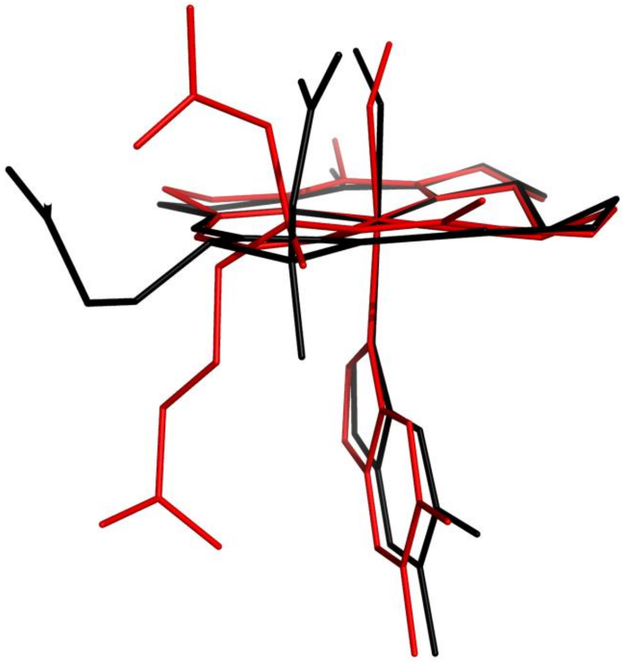

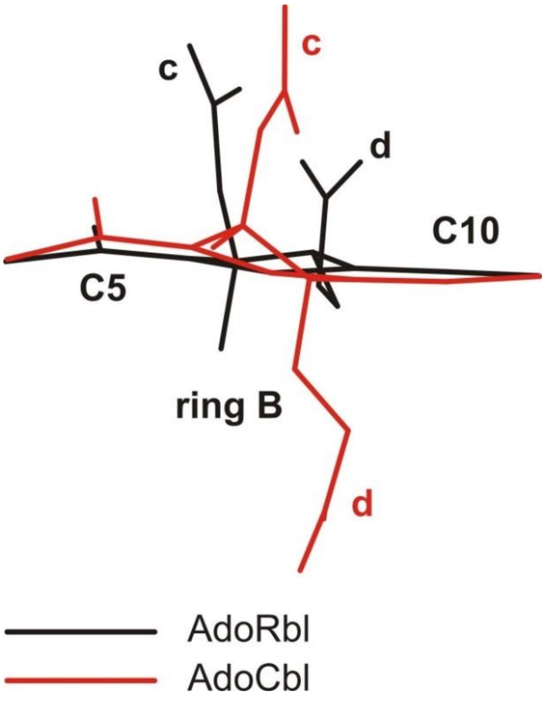

Figure S8. Top. Superposition of 'stick-models' of the core moieties of **AdoRbl** (black) and **AdoCbl** (red trace), highlighting the strongly differing side chain arrangements of the ring B section. **Bottom**. Comparison of superimposed structures of the ring B moieties of **AdoRbl** and **AdoCbl**, depictting the reversed ring conformation and the strongly differing side chain ar-rangements.

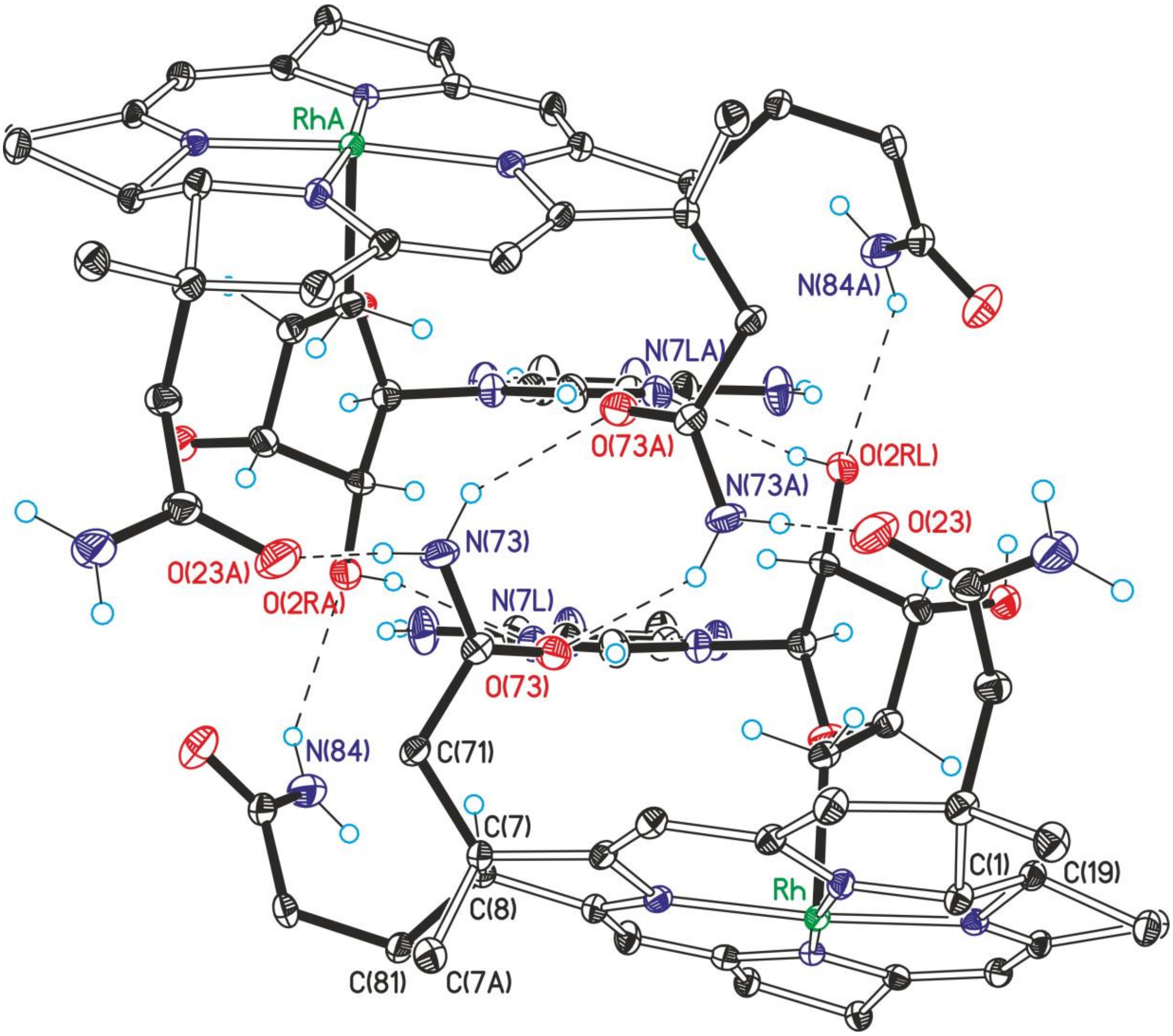

Figure S9. Structural details of the dimerization interface between two **AdoRbl** molecules in the crystal (for clarity only the core sections and functional groups at the interface are depicted, and the nucleotide loop is omitted). The C_2-symmetric arrangement of H-bonding and π-stacking interactions of the adenosyl group and of a, c and d-side chain moieties of the two **AdoRbl** molecules are highlighted (ORTEP-models with color coded heteroatoms and H-bonds drawn as dashed lines); see Figure S5 for atom numbering for **AdoRbl**.

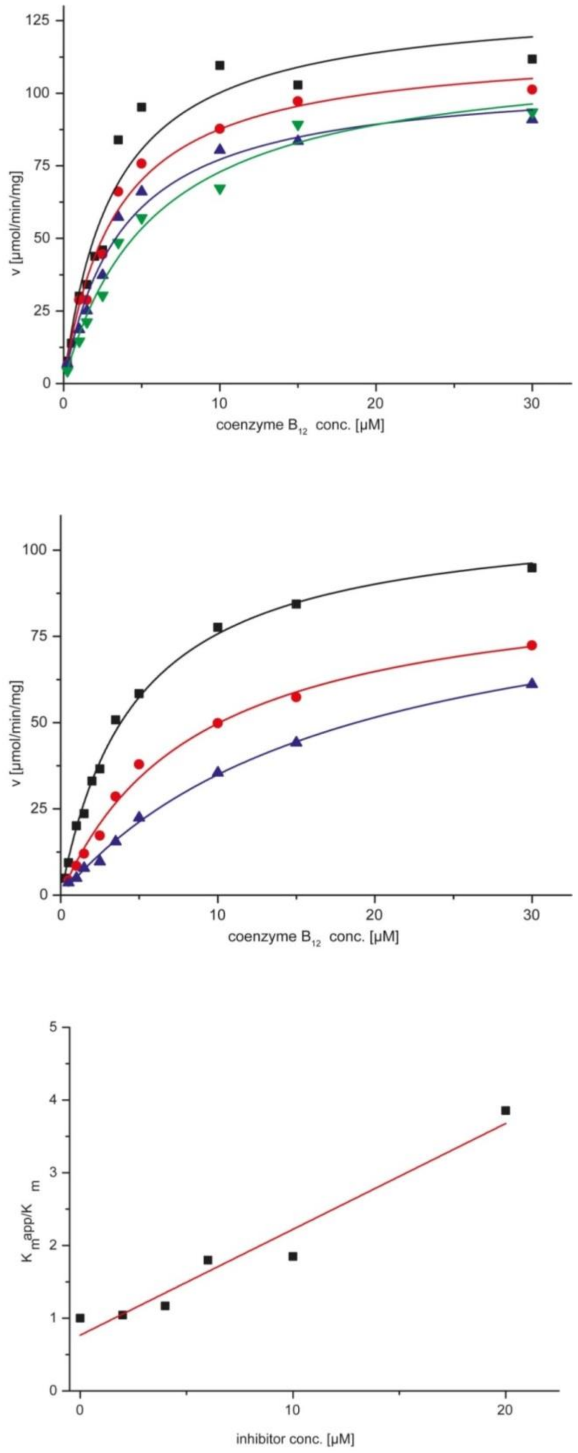

Figure S10. Top. Michaels-Menten kinetics of the diol dehydratase activity assay with different concentrations of adenosylrhodibalamin (**AdoRbl**), experiment 1, black: 0 µM (K_m = 3.2 µM; V_{max} = 132 µmol/min/mg), red: 2 µM ($K_m$app = 3.3 µM; V_{max}app = 117 µmol/min/mg), blue: 4 µM ($K_m$app = 3.7 µM; V_{max}app = 106 µmol/min/mg), green: 6 µM ($K_m$app = 5.7 µM; V_{max}app = 115 µmol/min/mg). **Middle.** Experiment 2, black: 0 µM **AdoRbl** (K_m = 4.7 µM; V_{max} = 111 µmol/min/mg), red: 10 µM **AdoRbl** ($K_m$app = 8.7 µM; V_{max}app = 93 µmol/min/mg), blue: 20 µM **AdoRbl** ($K_m$app = 18.1 µM; V_{max}app = 98 µmol/min/mg). **Bottom.** Plot of $K_m$app/K_m versus inhibitor concentration describing the inhibitory effect of adenosylrhodibalamin (**AdoRbl**).

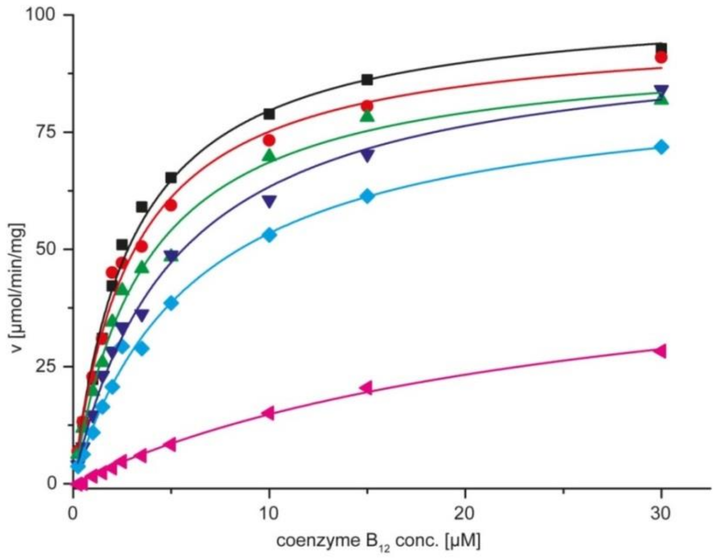

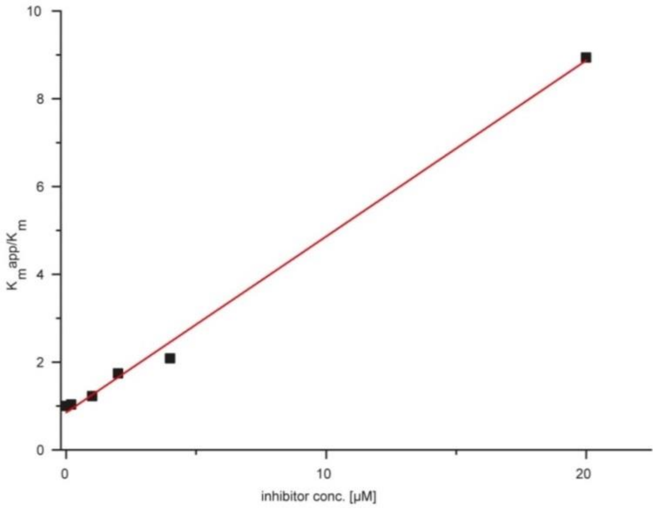

Figure S11. Top. Michaels-Menten kinetics of the diol dehydratase activity assay with different vitamin B_{12} (**CNCbl**) concentrations, experiment 1, black: 0 µM (K_m = 3.0 µM; V_{max} = 103 µmol/min/mg), red: 0.2 µM ($K_m$app = 3.0 µM; V_{max}app = 100 µmol/min/mg), green: 1 µM ($K_m$app = 3.6 µM; V_{max}app = 94 µmol/min/mg), purple: 2 µM ($K_m$app = 5.2 µM; V_{max}app = 96 µmol/min/mg), blue: 4 µM ($K_m$app = 6.2 µM; V_{max}app = 86 µmol/min/mg), purple: 20 µM ($K_m$app = 27 µM; V_{max}app = 54 µmol/min/mg). **Bottom.** Plot of $K_m$app/K_m versus inhibitor concentration describing the inhibitory effect of vitamin B_{12} (**CNCbl**), linear fit (red, y = 0.401 * x + 0.853).

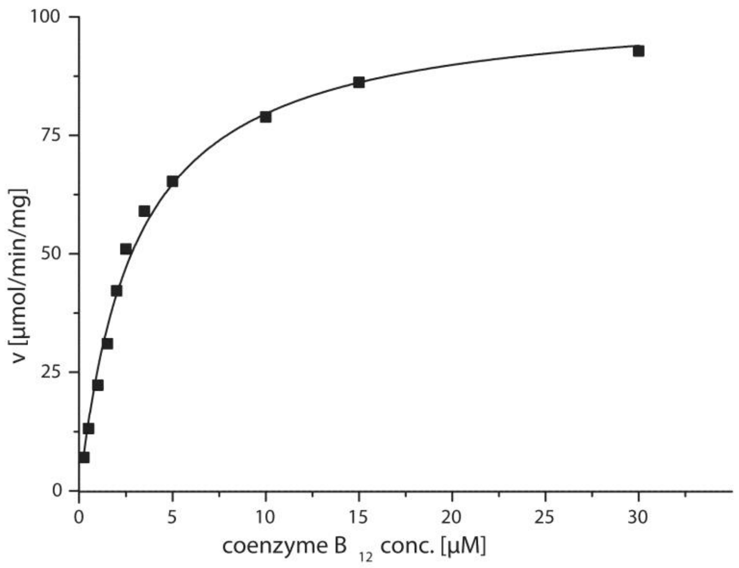

Figure S12. Michaels-Menten kinetics of the diol dehydratase activity assay with adenosylco-balamin (**AdoCbl**) (Km = 3.0 µM; Vmax = 103 µmol/min/mg).

References

1 Perrone, P. *et al.* First Example of Phosphoramidate Approach Applied to a 4‘-Substituted Purine Nucleoside (4‘-Azidoadenosine): Conversion of an Inactive Nucleoside to a Submicromolar Compound versus Hepatitis C Virus. *J. Med. Chem.* **50**, 5463-5470 (2007).

2 Eschenmoser, A. Vitamin-B_{12} - Experiments Concerning the Origin of Its Molecular-Structure. *Angew. Chem. Int. Ed.* **27**, 5-39 (1988).

3 Shibata, N., Masuda, J. Tobimatsu, T,, Toraya, T. Suto, K., Morimoto, Y., Yasuoka, N., A new mode of B_{12} binding and the direct participation of a potassium ion in enzyme catalysis: X-ray structure of diol dehydratase. *Structure* **7**, 997-1008 (1999).

4 Roessner, C. *et al.* Genetically engineered synthesis of precorrin-6x and the complete corrinoid, hydrogenobyrinic acid, an advanced precursor of vitamin B_{12}. *Chemistry & Biology* **1**, 119-124 (1994).

5 Otwinowski, Z. & Minor, W. Processing of X-ray diffraction data collected in oscillation mode. *Meth. Enzymol., Macromol. Crystallogr., Pt A* **276**, 307-326 (1997).

6 Sheldrick, G.M. SHELXL-97, a program for the refinement of crystal structure from diffraction data (University of Göttingen, Göttingen, 1997).

7 Toraya, T., Ushio, K., Fukui, S. & Hogenkamp, P. C. Studies on the mechanism of the adenosylcobalamin-dependent diol dehydrase reaction by the use of analogs of the coenzyme. *J. Biol. Chem.* **252**, 963-970 (1977).

8 Ascenzi, P., Ascenzi, M. G. & Amiconi, G. Enzyme competitive inhibition. Graphical determination of Ki and presentation of data in comparative studies. *Biochem. Education* **15**, 134-135 (1987).
